# Environmental reservoirs account for high levels of carbapenem resistance genes in wastewater

**DOI:** 10.1128/spectrum.01737-25

**Published:** 2026-01-07

**Authors:** Melissa K. Schussman, Shuchen Feng, Angela Schmoldt, Melinda J. Bootsma, Kieyarrah Dennis, Kayley H. Janssen, Sandra L. McLellan

**Affiliations:** 1 School of Freshwater Sciences, University of Wisconsin-Milwaukee 543317, Milwaukee, Wisconsin, USA; 2 Wisconsin State Laboratory of Hygiene, University of Wisconsin-Madison 37360 , Madison, Wisconsin, USA; Duke University, Durham, North Carolina, USA

**Keywords:** carbapenemase, sewage, wastewater surveillance, antimicrobial resistance

## Abstract

Wastewater surveillance has moved to the forefront as a practical and informative public health tool for assessing viral and bacterial pathogens in the human population; however, the usefulness for tracking antimicrobial-resistance genes (ARGs) of clinical concern is not straightforward given the large reservoir of free-living bacteria within sewer systems. In this study, we examined six high-priority carbapenem resistance gene targets and common host organisms in wastewater collected from two treatment plants. Polymerase chain reaction (PCR)-based assays demonstrated an extremely high abundance of *bla*_KPC_, *bla*_OXA-24/40_, and *bla*_OXA-48_ with mean concentrations as high as 3.55E+06 copy number (cn)/L for *bla*_KPC_. In addition, *bla*_VIM_ and *bla*_IMP_ were detectable in ~99% of samples at lower levels of ~1.5E+05 cn/L and 1.0E+04 cn/L, respectively. The target *bla*_NDM_ was readily detected but quantifiable in only 49% of samples with levels of ~1.0E+03 cn/L. Each PCR target showed one to two dominant sequence types that matched exactly to reported clinical strains, indicating that high levels could not be attributed to non-specific amplification. Select ARG targets showed moderate-to-weak correlations to human fecal markers and *Klebsiella pneumoniae* or *Escherichia coli*, whereas *bla*_OXA-24/40_ most closely correlated to *Acinetobacter baumannii*. The two treatment plants showed different dynamics, suggesting that the inherent characteristics of the individual conveyance systems influence concentrations. Culture on selective media revealed an abundance of *Aeromonas* in addition to sporadic *Enterobacteriaceae* carrying *bla*_KPC_. Understanding the ecological dynamics of bacteria harboring ARGs will be important for understanding reservoirs and interpreting wastewater surveillance data.

**IMPORTANCE:**

Wastewater surveillance for carbapenem-resistant bacteria has been proposed as a potentially valuable tool for assessing the human burden, but their distribution in environmental reservoirs is not well understood. Untreated sewage contains a high density of resident organisms mixed with human inputs, which are transported through the complex environment of sewer conveyance systems. In addition to resident microbial community members, organisms seeded into the system from the human microbiome have the potential to grow. This work shows that environmental bacteria may be a significant source of carbapenem resistance genes collected from sewer systems, making wastewater surveillance data difficult to interpret or use for public health actions. More knowledge is needed to unravel the ecology of these systems and identify targets for surveillance that are meaningful to clinicians. This work sheds light on the complex dynamics and confounding factors for antimicrobial-resistance gene wastewater surveillance, which will improve the interpretation of wastewater surveillance data.

## INTRODUCTION

Carbapenems possess broad-spectrum antibacterial activity and are unaffected by most β-lactamases and extended-spectrum β-lactamases (1), which make carbapenem-based antibiotics one of the most reliable drugs for treating bacterial infections worldwide (1, 2). However, antimicrobial resistance has emerged over the last 20 years, including in *Enterobacteriaceae*, which are among the most common human pathogens (3). Carbapenemase-producing organisms’ predominant mechanism of resistance involves the production of enzymes that cleave the carbapenem moiety in the antibiotic structure. Among the most effective in carbapenem hydrolysis and global abundance are KPC, IMP, NDM, VIM, and OXA-48 types, which consist of multiple variants (4–9). Furthermore, carbapenem resistance is commonly associated with the accumulation of additional resistance mechanisms for other antimicrobial classes (10). As a result, in 2017, the World Health Organization recognized fighting carbapenem resistance as a global priority in the highest priority category (11).

Wastewater monitoring holds promise as a viable surveillance method because it can provide information about carriage among an entire population while minimizing the effort, expense, and privacy concerns of clinical testing (12, 13). Specifically for antimicrobial resistance surveillance, wastewater offers the potential to rapidly pinpoint epicenters for evolution and spread while offering expansive data sets to identify drivers, predict trends, and inform health officials on which antibiotics could prove most effective for the treatment of resistant pathogens (13). In recent years, wastewater surveillance has been proposed as a means to quantify the abundance of antimicrobial-resistance genes (ARGs) within a population (14), but the influence of environmental and infrastructure variables on bacteria that carry ARGs requires further exploration. Current reports on carbapenem resistance surveillance indicate that while ARGs are readily detected in wastewater, these genes cannot be tied to healthy versus sick patients, or even human origin (15, 16). And so, many reports suggest that wastewater surveillance of ARGs cannot be reliably performed using traditional methods used for viral detection (i.e., filtering, extracting, and analyzing DNA via polymerase chain reaction [PCR]), and may require a more specialized and complicated work-up, including culture and sequencing (17–19) to understand the virulence, pathogenicity, and complete diversity of an associated ARG, rather than just detection of an ARG (20–22).

DNA encoding ARGs have been frequently detected in high concentrations in raw wastewater influent and hospital wastewater (23–25). However, the abundance and diversity of the detected ARGs have varied significantly between locations and even within different phases of treatment within wastewater treatment plants (WWTPs) (12). The presence of ARGs is likely in part due to human fecal inputs, which are of high interest for wastewater surveillance, though it is not clear how much of the signal is directly related to human carriage versus environmental occurrence (26). PCR-based assays for ARGs have been designed to confirm clinical isolations (27–30) and have been adapted to assess concentrations in wastewater (20, 31), but PCR testing alone does not identify the organisms or the specific sequence of the ARG, leading to questions on its origin and relevance to human health. Human fecal bacteria only account for approximately 15%–20% of the sewage influent community organisms (15), and studies have shown that as sewage moves downstream, the nonfecal portions of influent increase and are dominated by a high abundance of taxa distinct from the human fecal microbiome (32). Several of the common hosts of carbapenem-resistant ARGs, including *Acinetobacter*, *Klebsiella*, *Enterobacter*, and *Escherichia coli,* appear to be enriched in wastewater compared to human fecal samples (33), suggesting a non-linear relationship between detected ARGs and human inputs. Furthermore, microbial communities in the sewer conveyance system are exposed to many parameters that can impact their natural abundance, including temperature, flow, antimicrobial agents, chemicals, and heavy metals. These can create conditions that favor the survival or proliferation of specialized organisms, thereby potentially increasing the detectable concentration of ARGs (34, 35).

In this study, we quantified ARGs encoding clinically relevant carbapenemases across 122 samples from two WWTPs using digital droplet PCR (ddPCRs). We further employed amplicon sequencing of select samples to confirm our assays were quantifying the desired targets, and 16S rRNA gene sequencing to assess the microbial community and relative abundance patterns for taxa that typically carry these genes. Overall, the very high levels of some ARGs and enrichment of the taxa that carry these genes from conveyance system sites to the WWTPs suggest ARG levels are not directly related to human inputs. Warmer temperatures increased ARGs typically associated with *Enterobacteriaceae* and decreased ARGs carried by *Acinetobacter,* but the strength of these relationships differed by WWTP, which varied in configuration, travel times, and flow dynamics. Culture of wastewater on selective media revealed a high abundance of *Aeromonas* and *Enterobacteriaceae* (but not *E. coli* or *Klebsiella pneumoniae*), demonstrating a dominance of non-clinical organisms in wastewater. The extent to which wastewater surveillance can be used to assess carriage or infection with carbapenem-resistant bacteria in human populations needs to consider the ecology of these organisms in the complex sewer environment.

## RESULTS

### Sampling strategy and targets

This study examined six carbapenem resistance gene targets (Table 1) in wastewater samples collected over 1 year from two WWTPs that collectively service the Milwaukee metropolitan area in Wisconsin. The plants receive waste from multiple hospitals and serve approximately 500,000 people each, but have different travel times and inputs (Table 2). Samples from April and August were compared to examine differences across seasons as a proxy for temperature. Furthermore, monthly, weekly, and daily samples were analyzed to determine temporal fluctuations and understand the variability in the system.

**TABLE 1:** ARG genes and common host organisms in GenBank detected by the study assays^*a*^

Gene	Type of carbapenemase	No. of variants aligned	No. with primer mismatch^*b*^	No. with probe mismatch^*b*^	Gene target	Common host organisms^*c*^
*Klebsiella pneumonia*e carbapenemase	Ambler class A beta-lactamase (β-lactamase)	222	5	0	*bla*_KPC_	*Klebsiella pneumoniae*
Oxacillinase 24/40-like group	Class D β-lactamases (CHDLs)	14	0	6	*bla*_OXA-24/40_	*Acinetobacter* spp.
Oxacillinase 48-like group	Class D β-lactamases (CHDLs)	66	1	3	*bla*_OXA-48_	*Klebsiella pneumoniae*
Verona integrin-encoded MBL	Class B Metallo-β-lactamases (MBL)	88	0	6	*bla*_VIM_	*Pseudomonas* spp.
Imipenemase MBL	Class B β-lactamases	100	34	71	*bla*_IMP_	*Pseudomonas* spp.
New Delhi MBL	Class B β-lactamases	74	0	3	*bla*_NDM_	*Klebsiella pneumoniae* and *Escherichia coli*

^*a*^ Variant gene sequences were aligned to the ddPCR assays to determine specificity. All sequences used in the alignments were obtained from the NCBI Reference Gene Catalog.

^*b*^ Variants with one of more mismatch based on BLAST search of sequences obtained from the Reference Gene Catalog (https://www.ncbi.nlm.nih.gov/pathogens/refgene/#).

^*c*^ Multiple organisms carry these genes, but only the most commonly reported, defined by occurrence in the NCBI database are listed.

**TABLE 2:** Description and details of WWTPs sampled in this study^*a*^

WWTP	Population	Approx. period of development	Avg occupied hospital beds^*b*^	Flow (MGD)	BOD (mg/L)	Avg travel time (hours)	System type	No. of samples
Jones Island (JI)	470,007	Pre-1920–1950	934	39–223 (91)	54–360 (214)	7.5	37% combined	36 + 25 daily
South Shore (SS)	615,934	1920–present	788	34–262 (88)	120–680 (314)	22	separate	36 + 25 daily

^*a*^ Flow and biological oxygen demand (BOD) are reported as a range of minimum to maximum, with daily average (in parentheses).

^*b*^ Approximate estimate from the major hospitals (data provided by the Wisconsin Department of Health Services) over the time period of this study.

### Concentrations of high-priority carbapenem resistance gene targets in wastewater

**Fig 1:**
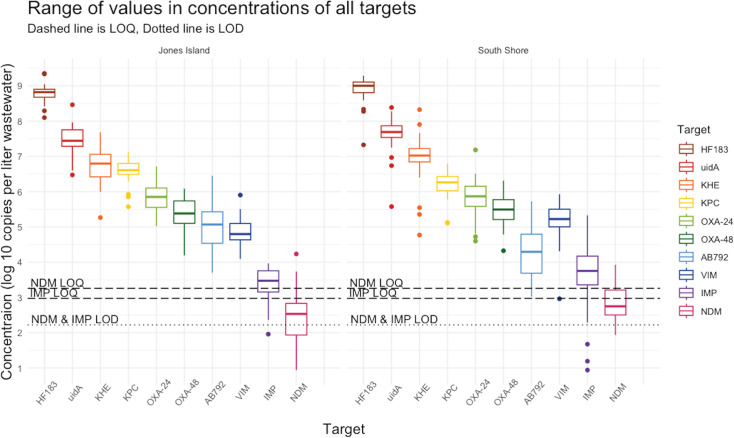
Box plots of log10 transformed concentrations per liter of human markers and ARG targets in JI and SS WWTP across 2022–2023. The box frames the interquartile range (IQR), containing a line in the middle that represents the median. Whiskers extending above and below the box indicate variability outside Q1 and Q3. The minimum/maximum whisker values are calculated as Q1/Q3 ± 1.5 * IQR. All data points outside of the box and whiskers are outliers in the data, *n* = 36 per WWTP.

We found all six carbapenem ARGs were readily detected in both WWTPs (Fig. 1). The genes *bla*_KPC_, *bla*_OXA-24/40_, *bla*_OXA-48_, and *bla*_VIM_ were quantified in 100% of the samples with mean concentrations up to 3.55E+06 copies per liter (cn/L) for *bla*_KPC_, 1.11E+06 cn/L for *bla*_OXA-24/40_, 4.08E+05 cn/L for *bla*_OXA-48_, and 1.58E+05 cn/L for *bla*_VIM_. The target *bla*_IMP_ was detected in the majority of samples, but at concentrations 1–2 orders of magnitude lower (1.13E+04 cn/L), with 2.7% of samples falling below the limit of quantification (BLQ), and 1.3% below the limit of detection (BLD). The target *bla*_NDM_ had quantifiable levels less frequently (BLQ in 51% of the samples and BLD in 4.1% of samples), and relatively low average concentrations (1.05E+03 cn/L).

### Amplicon sequencing of carbapenem resistance gene targets

**Fig 2:**
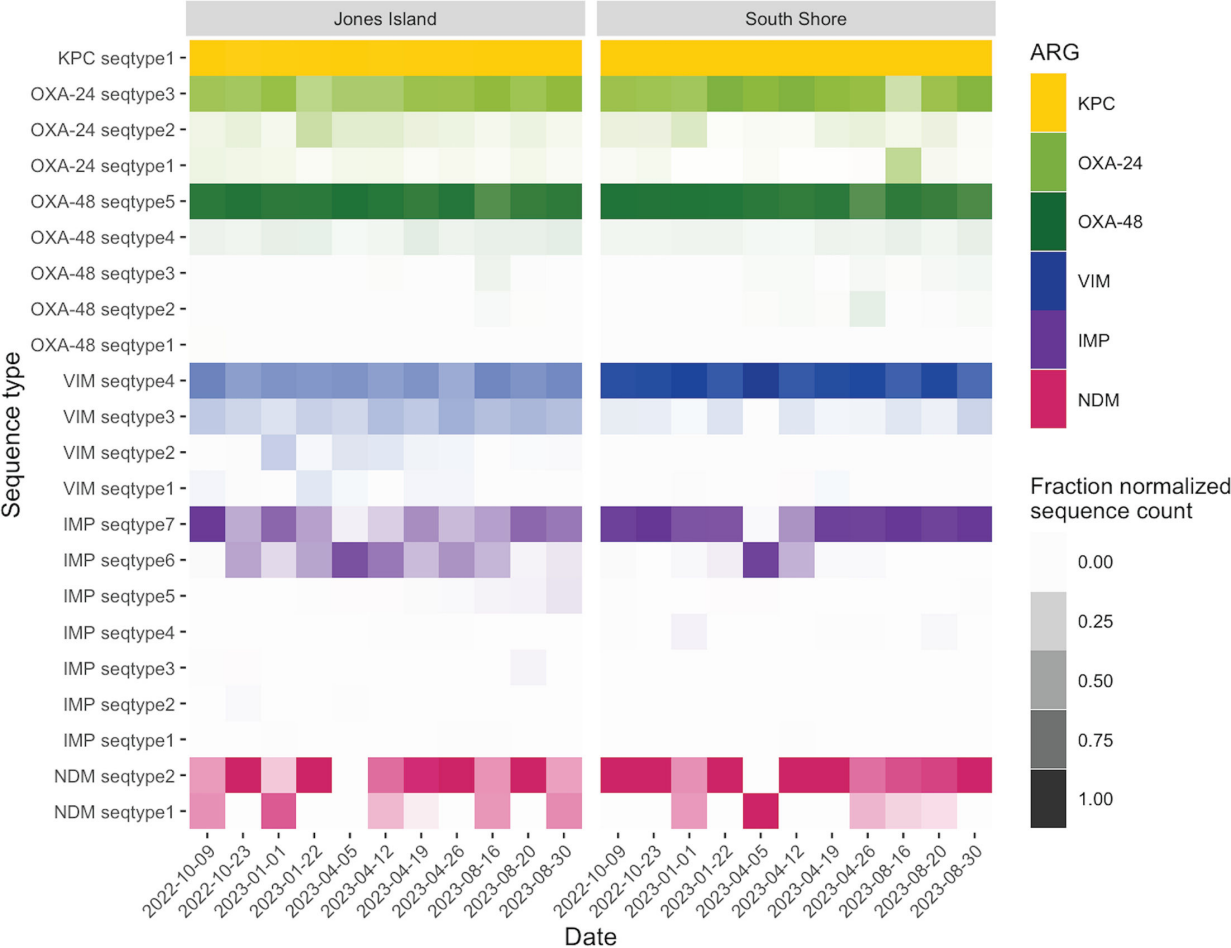
Distribution of amplicon sequencing types across wastewater samples collected in October, January, April, and August. Sequence types represent all specific sequences (>99% of the data set). Most of the carbapenemase ARGs showed one dominant sequence type with minor sequence types. *bla*_KPC_ has only one sequence type, while *bla*_VIM_ and *bla*_IMP_ illustrated the most diversity in the amplicon region.

NCBI Basic Local Alignment Search Tool (BLAST) (36) was used to verify the primer specificity, variants detected, and the presumptive host organisms carrying these genes in each set of assay primers and probes (Table 1; Table S1). Amplicons from each of the six ARG targets were then sequenced to confirm the product and assess the diversity of genes present. The vast majority (>99%) of the sequenced amplicons were specific for their gene target, with minimal nonspecific amplification. For *bla*_KPC_, there was only one sequence type, which was expected as the assay amplifies a highly conserved region of the gene. The remaining targets had between two and seven sequence types (Fig. 2). Both WWTPs had similar profiles of the ARG sequence types, with the exception of a more even distribution of sequence types 1 and 2 for *bla*_VIM_ and *bla*_IMP_ in JI WWTP compared to SS WWTP.

Interestingly, the *bla*_OXA-24/40_ and *bla*_OXA-48_ ddPCR assays demonstrated a multimodal amplitude of the droplet clouds, indicating that multiple variants were being amplified (Fig. S1) (37). Amplicon sequencing data confirmed this phenomenon (Fig. 2), with both assays containing at least one relevant mismatch on the probe and/or primers that impacted the amplitude of the cloud. The amplicon sequencing showed one dominant sequence type and a collection of minor sequence types, which was consistent with the proportion of droplets within the individual clouds at different amplitudes.

An additional BLAST search was used to assess the most common organisms in the NCBI nucleotide database that were associated with amplicon sequence types. We found the proportion of BLAST hits for each amplicon sequence type matched what was expected based on assessing only the primer and probe matches (Table S1), further confirming our assays amplified the intended target sequences.

### Enrichment of taxa associated with carbapenem ARGs in downstream samples

We hypothesized that high levels of ARGs are present in wastewater because genes are detected from not only human fecal matter but also from bacteria that accumulate or even grow in the conveyance system infrastructure. Using microbial community 16S rRNA data from a previous study in this same system (38), we examined the organisms most frequently associated with ARGs in upstream sites within the conveyance system and downstream at the WWTP to determine whether there was an increase in relative abundance without an increase in human microbiome fecal markers, which could be an indication of growth and replication in the pipes. For human fecal markers, we used a single amplicon sequence variant (ASV) that maps to the HF183 (Bacteroides_B) marker (39) and also a number of Blautia_A ASVs since members of this genus have been found to be human-specific (40).

As expected, we found that the human-derived ASVs decreased in relative abundance from upstream to the WWTP by threefold to fourfold, except in samples at JI WWTP, where Bacteroides_B was at levels similar to conveyance system sites (Table S2). *Escherichia* (which was represented as a single ASV) also decreased in relative abundance from the conveyance system to the plant; however, *Enterobacter*, *Klebsiella*, *Shewanella,* and ASVs that could only be resolved to the family *Enterobacteriaceae* (which may represent additional *Escherichia*) increased up to ~6-fold for certain genera. A second *Bacteroides* ASV marker that is more specific to conveyance system pipes than the human fecal microbiome (41) demonstrated a 2-fold increase, and *Acinetobacter*, *Aeromonas*, and *Arcobacter*, which are three of the most abundant taxa in WWTP samples, increased ~2- to 6-fold, with a lesser amount of enrichment in JI WWTP compared to SS WWTP.

### Influence of flow at the WWTP on carbapenem ARG concentrations

To assess the influence of flow without the added complications of changing temperature, we first examined the relationship of flow using daily samples collected over 25 consecutive days, when temperatures were consistent. We compared concentrations of seven targets (four ARG targets, two human markers, and *E. coli*) between high-flow days (upper 1/3 of flow measurements) and low-flow days (lower 1/3 of flow measurements). At JI WWTP, we found significantly lower concentrations on high-flow days for human markers. *E. coli* and two of the four ARG/host organism markers, whereas at SS WWTP, only the human marker concentrations were significantly lower (Fig. S3). When examining the entire year using this same approach, which had more variable flow, none of the targets were significantly lower in high-flow days compared to low-flow days (Fig. S4), which may suggest that the growth or decay of targets influences final concentrations to a similar degree as dilution effects of high flows.

### Influence of temperature (seasonality) on carbapenem ARG concentrations and specific taxa within the microbial community in WWTP samples

We further examined the correlation of ARG and host organism concentrations to temperature. Prior studies in this system demonstrated a clear seasonal cycling pattern over multiple years for several of the organisms targeted in this study (38). To account for the large range of flow exhibited by JI WWTP resulting from its partially combined system, we normalized all target concentrations to flow. Multiple ARG targets illustrated a positive correlation to temperature (Table 3). The human fecal marker HF183 was expected to decrease in warm conditions, and we noted a significant negative correlation (*P* < 0.05) in SS WWTP, but not JI. The *bla*_OXA-24/40_ and *A. baumannii* (AB792) markers also exhibited a significant negative correlation (*P* < 0.05) to temperature in SS WWTP. *E. coli* and *K. pneumoniae* (KHE) were significantly higher in warmer temperatures in JI WWTP (*P* < 0.05), as were *bla*_KPC_ and *bla*_OXA-48_. But these same high-abundance ARGs and their presumptive host organisms did not show clear temperature patterns in SS WWTP.

**TABLE 3:** Spearman’s correlation of flow-normalized organism-specific marker and ARG concentrations to temperature in Jones Island and South Shore WWTPs

Marker	Jones Island		South Shore	
	*R* value^*a*^	*P*-value^*a*^	*R* value	*P*-value
HF183	−0.15	0.39	**−0.37**	**0.027**
Lachno3	−0.24	0.33	−0.33	0.15
*K. pneumoniae*	**0.66**	**<0.0001**	0.22	0.19
*E. coli*	**0.52**	**0.0014**	0.32	0.055
*bla*_KPC_	**0.38**	**0.022**	−0.0069	0.97
*bla*_OXA-48_	**0.71**	**<0.0001**	0.17	0.32
*A. baumannii*	−0.074	0.67	**−0.38**	**0.022**
*bla*_OXA-24/40_	−0.23	0.17	**−0.59**	**0.00019**
*bla*_VIM_	**−0.39**	**0.018**	−0.3	0.071
*bla*_IMP_	−0.23	0.18	0.12	0.47
*bla*_NDM_	0.2	0.24	**0.49**	**0.0027**

^*a*^ Significant values are shown in bold.

A Wilcoxon test was performed to compare all marker concentrations (normalized to flow and population) to temperature using samples (*n* = 8) from April (low temp) and August (high temp), which have been established as the months with the most disparate temperatures in this system (38, 42). We found similar results to the overall correlations of the entire year time series with temperature, with *E. coli*, KHE, and *bla*_KPC_ demonstrating significantly increased concentrations (*P* < 0.05) in August samples at JI WWTP, with only *E. coli* increasing in warmer temperatures in SS WWTP. In contrast, *bla*_OXA-24/40_ was significantly decreased in August compared to April.

We found not only that select host organism markers and ARGs were positively correlated to temperature, considering the ddPCR quantitative results, but the relative abundance of specific microbial community members also shifted with temperature (Table S3) based on 16S rRNA microbial community data. *Klebsiella* and *Escherichia* in the community had a significant positive relationship with temperature across both JI and SS WWTPs. *Acinetobacter* and *Pseudomonas* were among the more dominant taxa in the community, and both had a weak but significant negative relationship with temperature. These data offer a second verification of temperature effects.

### Relationship among ARGs, presumptive host organisms, and human fecal markers

Finally, we looked at ARGs and relationships to their presumptive host organisms (based on NCBI BLAST results) or human fecal markers over the time series to examine the variability and the effect of normalization to HF183. Our hypothesis was that if the source of the ARG signals was primarily from human inputs, we would find a strong correlation to the HF183 human fecal marker. We found these relationships were dependent on the WWTP site. The ARGs most closely associated with *E. coli* and *Klebsiella* host organisms had moderate to low correlations to the HF183 marker. Each respective host organism and the strength of these relationships were generally weaker in JI WWTP compared to SS WWTP. Overall, JI WWTP demonstrated more variability in the ratios of ARGs to human and host organism markers compared to SS WWTP. The target *bla*_OXA-24/40_ was most closely correlated to *A. baumannii*, but notably, the overall *bla*_OXA-24/40_ levels were an order of magnitude higher than the *A. baumannii* marker. The ARGs *bla*_VIM_, *bla*_IMP_, and bla_NDM_ showed no clear patterns of correlations to human markers or host organisms, with a few sporadic weak correlations, presumably due to the wide variety of organisms that carry these genes. Alternatively, weak correlations with the more rare targets, specifically *bla*_NDM_, may be a result of the high number of samples that were detected, but not quantified.

### Cultured carbapenem-resistant organisms from untreated wastewater

To further analyze the relationship of ARGs to their prospective host organisms, we obtained 51 isolates distributed across three colony morphologies (presumptive *E. coli*, *Klebsiella-Citrobacter-Enterobacter*, and *Acinetobacter*) on selective media for KPC-producing organisms and performed 16S rRNA gene sequencing. We also screened the individual isolates for the ARG and host organism markers using PCR (see Materials and Methods and Fig. S6). PCR results showed that only 20 isolates contained *bla*_KPC_ (*n* = 18) or *bla*_OXA-24/40_ (*n* = 2) (Table S4), and none of these were positive for the host markers *E. coli*, KHE, or *A. baumannii*. Further 16S rRNA gene sequencing analysis demonstrated that the majority of *bla*_KPC_-carrying organisms were *Aeromonas*, which varied significantly from what was expected based on NCBI databases (Table 4). The remaining colonies (*n* = 31) were negative for all of the ARGs tested and were also primarily *Aeromonas* or *Pseudomonas,* with some sporadic *Enterobacteriaceae* detected.

**TABLE 4:** Expected versus observed ARG gene frequency detected from colonies grown and tested from 20 to 30 μL raw wastewater on CHROMagar KPC plates

BLAST of primers and probe		Analyzed colonies^*a*^	
Taxa	% frequency	Taxa	% frequency
*bla*_KPC_			
*Klebsiella pneumoniae*	65	*Aeromonas*	83.3
*Escherichia coli*	7	*Citrobacter*	5.6
*Pseudomonas aeruginosa*	4	*Enterobacteriaceae*	5.6
*bla*_OXA-24/40_			
*Acinetobacter baumannii*	93	*Acinetobacter*	50
*Acinetobacter nosocomialis*	4	*Pseudomonas*	50
*Acinetobacter haemolyticus*	2		
*bla*_OXA-48_			
*Klebsiella pneumoniae*	55	No detections	
*Escherichia coli*	19		
Uncultured bacterium	4		
*bla*_VIM_			
*Pseudomonas aeruginosa*	43	No detections	
*Klebsiella pneumoniae*	11		
*Enterobacter hormaechei*	4		
*bla*_IMP_			
*Pseudomonas aeruginosa*	24	No detections	
*Escherichia coli*	15		
*Enterobacter hormaechei*	14		
*bla*_NDM_			
*Escherichia coli*	32	No detections	
*Klebsiella pneumoniae*	28		
*Pseudomonas aeruginosa*	4		

^*a*^ *bla*_KPC_ was detected in 18 isolates, *bla*_OXA-24/40_ was detected in 2 isolates, and no other genes were detected among any of the isolates (*n *= 33).

## DISCUSSION

The very high levels of multiple carbapenem ARGs in wastewater in our study suggest that environmental reservoirs of organisms carrying these genes are present in sewer conveyance systems. This agrees with other studies that demonstrated similarly high levels of ARGs in community and hospital wastewater (23, 25, 26), and hospital sewage inputs did not influence the ARGs in overall community wastewater (26). The low levels of *bla*_NDM_ might suggest that organisms carrying NDM are not established, and long-term monitoring of levels within sentinel sites could document the accumulation of resident organisms. This complex community of potential hosts and ARGs makes wastewater surveillance data difficult to interpret for public health actions. Here, we explored the potential for conveyance systems to influence ARG levels and examined the association of high-abundance ARGs with their presumptive host organisms. We considered six ARG targets that are a high priority in clinical infections; three that are most often associated with *Enterobacteriaceae* found within the human microbiome (*bla*_KPC_, *bla*_OXA-48_, and *bla*_NDM_), one target that is found most often in *Acinetobacter*, an organism that occurs in the widely in the environment but also colonizes humans (*bla*_OXA-24/40_), and two that are in a wide range of organisms from both human and environmental origin (*bla*_VIM_ and *bla*_IMP_).

### Specificity of ARG assays

We first confirmed the amplicons matched the intended ARG targets, which rules out non-specific amplification as the source of high concentrations in wastewater for some of the targets. The genes *bla*_VIM_ and *bla*_IMP_ had the most diversity within the amplification region, consistent with the broad host range of organisms that include typical human microbiome members (i.e., *Enterobacteriaceae*) and environmental organisms (*Acinetobacter* spp. and *Pseudomonas* spp.), all of which have been implicated in clinical infections (43, 44).

Previous work with SARS-CoV-2 demonstrated that ddPCR was able to identify the proportion of positive droplets that are variants of the original target (37). In this study, using ddPCR instead of quantitative PCR (qPCR) provided the advantage of discerning mismatches in the primer or probe regions since these ARG targets appeared as diminished amplitude in the fluorescent signal (Fig. S1). The distribution of high- and low-amplitude signals showing dominant perfect matches and minor variants was confirmed by sequencing of the amplicons. While the amplitude of the cloud is affected, the overall concentration is not, since the lower amplitude droplets are counted as positives. Such minor variants could be underestimated or missed if quantified using qPCR (45).

### Propagation of organisms that carry carbapenem ARGs

Three separate findings support the conclusion that bacteria carrying ARGs have the potential to increase through growth in the conveyance system, particularly for *bla*_KPC_ and *bla*_OXA-48_. While several commonalities were found in the two WWTP systems, some results varied in magnitude or occurrence, highlighting the influence of system conditions on the survival and/or potential growth of bacteria harboring ARGs.

First, we found that the relative abundance of human-specific fecal anaerobes generally decreased from sampling sites in neighborhoods, while *Enterobacteriaceae, Aeromonas,* and *Acinetobacter* spp. increased. The shifts in relative abundance between the human fecal markers and the taxa that are known to carry carbapenemase ARGs are not sufficient to distinguish growth versus differential decay. It is possible that the human fecal markers decay more quickly than these organisms in transit. However, we saw very similar patterns of enrichment in both the SS and JI systems despite different travel times, while the loss of HF183 was negligible in JI (as fast as 4-h travel time) and reduced by twofold (200%) in the SS system (22-h travel time). In addition, the proportion of human fecal anaerobes, *Blautia,* and the HF183 marker was low compared to *Enterobacteriaceae* (38, 46), despite fecal anaerobes comprising a major portion of the fecal microbiome (47, 48). This suggests organisms carrying ARGs can be enriched in or have colonized the wastewater system (33).

Second, the very high levels of the ARGs themselves suggest standing reservoirs of these organisms, particularly for *bla*_KPC_. The target *bla*_KPC_ was approximately 1E6 cn/L in a sewershed of approximately 500,000 people, which would equate to 1.3E+09 copies per person per day, considering average flows for these WWTPs (Table 1). While we do not have an estimate of carriage in the general population or comprehensive shedding rate information for colonized individuals, detection in one study of known *bla*_KPC_ carriers and their contacts was less than 10% colonization (49). Detected *bla*_KPC_ concentrations were approximately an order of magnitude higher than what was found for SARS-CoV-2 levels in this same sewer service area at the height of the Omicron surge in the pandemic (37). Furthermore, in wastewater, *bla*_KPC_ and *bla*_OXA-48_ were detected at concentrations only two to three orders of magnitude lower, respectively, than HF183, which has been reported to be shed from humans at a mean concentration of 6E+10 copies per ng of feces (50).

Finally, there are significant positive correlations of *E. coli*, *KHE*, *bla*_KPC_, and *bla*_OXA-48_ to temperature in the JI system, indicating that warmer temperatures may help specific bacterial organisms grow or persist in the sewer system. Reports have shown that bacterial communities in wastewater systems across the United States are heavily impacted by temperature and follow seasonal patterns (38). While this study only encompasses a single year of sampling, potentially introducing seasonal and temporal bias by not capturing year-to-year variation or accounting for environmental anomalies, such as rain or weather events, past reports have also shown seasonal patterns of ARG abundance in untreated wastewater that could be influenced by ecological processes or temperature (26). Environmental conditions, such as temperature, have been shown to promote the horizontal gene transfer (HGT) of ARGs by enhancing the activity of integrases (51), which could have long-term implications for expanding reservoirs of clinically important ARGs. We did note similar trends in the SS system, but these were not significant. The higher BOD in the SS system in summer may indicate anaerobic conditions that did not favor the growth of *Enterobacteriaceae*. Only *bla*_NDM_ was positively correlated to temperature in the SS WWTP, but this is likely due to a larger number of samples that were quantifiable in summer months during low-flow conditions, which may have increased concentrations to detectable levels.

### ARGs common to *Acinetobacter* or *Pseudomonas* decreased in warm weather

In contrast to the increases in *Enterobacteriaceae* and their ARGs in warmer temperatures in the JI system, we found *bla*_OXA-24/40_ and *A. baumannii* had the opposite dynamics, demonstrating a negative correlation to temperature. This was particularly pronounced in the SS WWTP, which had a high BOD in warmer months, representing a more anaerobic environment. Both *Acinetobacter* and P*seudomonas*, the primary organisms found to carry *bla*_OXA-24/40_, are strict aerobes and therefore are unlikely to thrive in low oxygen conditions, especially in a system with long travel times. Overall, *bla*_OXA-24/40_ was at higher concentrations than *A. baumannii*, suggesting other host organisms carry *bla*_OXA-24/40_. The assay used in this study is specific for *A. baumannii* and does not detect *A. pittii*, *A. nosocomialis*, and *A. junni*, all of which have been reported to host *bla*_OXA-24/40_ in clinical samples (52). The BLAST results of the PCR amplicon for the ARG assays were based on the organisms deposited in NCBI GenBank (53), which may be overrepresentative of certain clinical strains and may not be reflective of the proportion of those organisms in conveyance systems. The targets *bla*_VIM_ and *bla*_IMP_ are carried in a variety of organisms, including *Acinetobacter* sp. and *Pseudomonas* spp., and we found that *bla*_VIM_ was also negatively correlated with temperature, but this trend was only significant in JI WWTP. We did not observe a clear temperature pattern for *bla*_IMP_, which may be due to the lower levels in the wastewater systems.

### ARG host abundance in wastewater conveyance systems greatly diverges from clinical patterns

The profile of isolates cultured from raw wastewater displayed a stark contrast between presumed host organisms in NCBI (Table 4; Table S4). The presumptive host organisms identified in NCBI BLAST bias clinically relevant strains. Because wastewater conveyance systems accommodate dense, complex bacterial communities, they offer a prime opportunity for the emergence and transfer of ARGs (54, 55). This may explain why correlations of ARGs to human markers were weak, namely in *bla*_IMP_ and *bla*_NDM_, despite their rarity in detection, which might suggest they are not established in the conveyance system. IMP- and VIM-like carbapenemases are often found within a gene cassette carried in class 1 integrons that within themselves are quite diverse and have been found in *Enterobacteriaceae* and non-fermenters such as *Pseudomonas aeruginosa* and *Acinetobacter* spp. (56). While these three genes were not detected in our isolates from our samples, *Pseudomonas* spp. and *Acinetobacter* spp. accounted for the two *bla*_OXA-24/40_ detections.

Strikingly, *bla*_KPC_ dissemination is commonly driven by mobile genetic elements such as the transposon Tn4401 and various plasmids that are highly adapted to *Enterobacteriaceae* hosts and do not spread as efficiently to non-*Enterobacteriaceae* Gram-negatives, and therefore is believed to be predominantly confined to *Klebsiella pneumoniae* and other *Enterobacteriaceae* (57), which are also the main organisms expected on ChromAgar KPC. However, this gene was detected primarily in *Aeromonas* spp. and could account for the very high levels in wastewater. Previous studies have published similar findings (58–60), with *bla*_KPC_ being detected on an IncU plasmid of *Aeromonas* (59). HGT is the primary mechanism responsible for the widespread distribution of ARGs located on plasmids, and so it is important to consider alternative hosts in the wastewater environment, which promotes HGT.

### Each WWTP displayed unique dynamics in ARG concentrations

**Fig 3:**
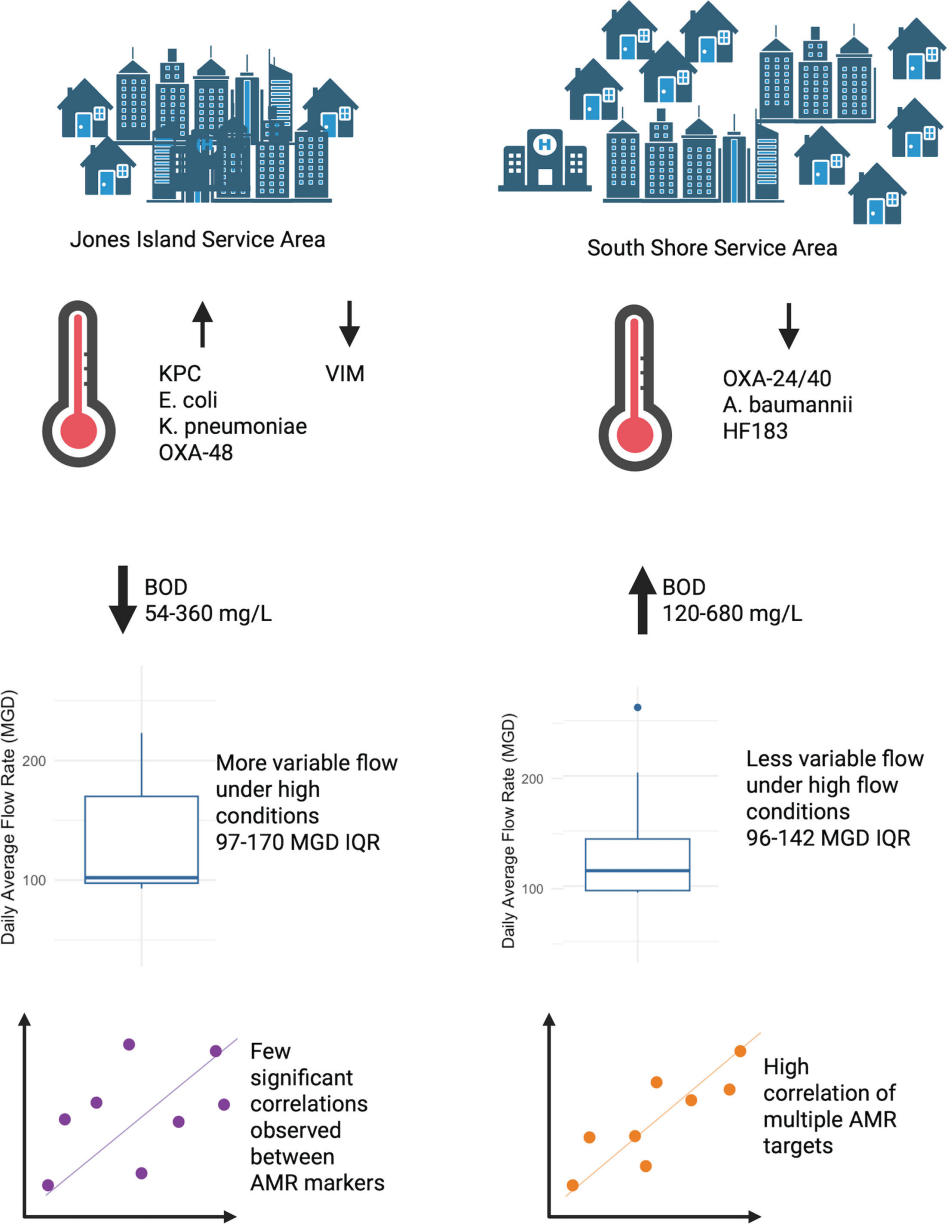
Differences in ARG and conveyance system dynamics in Jones Island and South Shore WWTPs.

While both WWTPs showed very high levels of high-priority ARGs, the contrasting dynamics in Fig. 3 highlights the large influence of each unique conveyance system on concentrations measured at each WWTP. Parameters such as flow and temperature have been shown to have an impact on viral targets measured in wastewater surveillance programs (42), and the relative abundance of bacterial taxa (26, 38), but little work has been done on the impact of wastewater parameters on ARG concentrations. Longer travel times in the SS system and higher BOD in summer appeared to affect the aerobic community members, and possibly the *Enterobacteriaceae,* which are facultative anaerobes.

Neither JI nor SS exhibited a significant difference in concentrations between high-flow and low-flow days when considering all samples across 2022–2023. This could be attributed to the low variance in flow at both WWTPs, which changed at most twofold to threefold. We hypothesize that there would be lower correlations of ARGs to the HF183 if non-fecal sources were contributing “out of sync” with the human fecal input. The low variability in flows to SS WWTP (Fig. S3 and S4) could account for the consistent, moderate correlations of all the ARGs with HF183 and their presumptive host organisms, suggesting a common delivery of human inputs and environmental reservoirs. In contrast, high variability of high-flow conditions in JI WWTP as a result of the partially combined sewer network could account for differing patterns of mobilization.

### Conclusions

The complexity of wastewater systems and reservoirs of organisms within pipes is likely driving concentrations of ARG targets more than human input. *Aeromonas*, *Acinetobacter*, and *Pseudomonas* are consistently among the most abundant microorganisms in untreated sewage (32, 38, 46). It is unclear if the host organisms harboring the ARGs were originally seeded from humans or arose from HGT within the system, and if they represent transient reservoirs or more stable standing stocks that constantly seed the system. The presumptive host organism markers demonstrated a low correlation to the ARGs, and sequencing of isolates confirmed that the majority of the detected ARGs were found in environmental organisms. This paper highlights the additional complexities of wastewater when analyzing bacterial targets, which may result in a need for wastewater-specific assays, instead of adaptation of clinical assays for wastewater applications. Further investigation is needed to determine whether the ARG signals are linked to pathogens and to examine the establishment and growth potential of these specific organisms. While measurements at the community level at the WWTPs provide a baseline, wastewater surveillance geared toward public health action for ARGs is more applicable at the facility level (61). Smaller-scale sampling minimizes but does not completely negate these complications, and ARG reservoirs in local pipes would need to be assessed (62). More specific assays, linkage between organisms and genes, and culture-based methods will be necessary to disentangle the ARG signals from different sources in wastewater surveillance efforts.

### Limitations

The results of this study are reliant on 1 year of testing. Further testing is needed to determine whether patterns of ARG in response to temperature are repeatable on an annual basis. Furthermore, isolation on selective media and 16S rRNA gene sequencing only captured the most abundant organisms, which was sufficient to demonstrate the large influence of *Aeromonas* on *bla*_KPC_ levels, and to suggest that *bla*_OXA-24/40_ is frequently detected in organisms other than *A. baumannii*. A comprehensive culture-based study is needed to verify these findings and to investigate the common host organisms for the other rarer targets in this study. We recommend further research, including broader sampling, isolation, and sequencing analysis to promote a comprehensive understanding of carbapenem resistance within the sewer network.

## MATERIALS AND METHODS

### Selection of antibiotic resistance gene targets

We chose six antibiotic-resistance gene (ARG) assay targets that are high-priority antimicrobial-resistant bacteria targets as they are characterized by the CDC to be both “urgent” and “serious” threats (63). Assay sequences and corresponding information are indicated in Table 5.

**TABLE 5:** Gene targets of various ARGs and host organisms examined in this study with corresponding Taqman ddPCR and qPCR assays^*a*^

Gene	Platform	Primer name	Sequence 5′−3′	Size	Source
*bla*_KPC_	ddPCR	KPC F	5′-GGCCGCCGTGCAATAC-3′	60 bp	Assay designed Kirby et al. (64) for detection in lysates made from gram-negative bacteria
		KPC R	5′-GCCGCCCAACTCCTTCA-3′		
		KPC *P*	5′-FAM/TGATAACGC/ZEN/CGCCGCCAATTTGT/IABkFQ-3′		
*bla*_OXA-40/24-like_, *bla*_OXA-48-like_ duplex	ddPCR	OXA-24 F	5′-GATGACCTTGCACATAACCG-3′	150 bp	Designed by Feng et al. (65) for detection in clinical isolates
		OXA-24 R	5′-CAGTCAACCAACCTACCTGTG-3′		
		OXA-24 P	5′-HEX/AGTAACACC/ZEN/CATTCCCCATCCACTTTT/IABkFQ-3′		
	ddPCR	OXA-48 F	5′-ACGGGCGAACCAAGCAT-3′	59 bp	Designed by Feng et al. (65) for detection in clinical isolates
		OXA-48 R	5′-GCGATCAAGCTATTGGGAATTT-3′		
		OXA-48 P	5′-FAM/TTACCCGCA/ZEN/TCTACC/IABkFQ-3′		
*bla*_VIM_	ddPCR	VIM F	5′-GTGAGTATCCGACAGTCARCGAAA-3′	142 bp	Designed by WI state lab of hygiene for the purpose of wastewater surveillance
		VIM R	5′-TCACCATCACGGACAATGAGACCA-3′		
		VIM P	5′-FAM/CYGATGGTG/ZEN/TTTGGTCGCATATCGCAAC/IABkFQ-3′		
*bla*_IMP_	ddPCR	IMP-1-F	5′-GGCTTAATTCTCGATCTATCCC-3′	113 bp	Designed Roguet and Schussman (66) for detection in clinical isolates
		IMP-1-R	5′-CTAGCCAATAGTTAACTCCGC-3′		
		IMP P	5′-HEX/GACGGTAAG/ZEN/GTTCAAGCCACAAAT/IABkFQ-3′		
*bla*_NDM_	ddPCR	NDM F	5′-CTGGGCGGTCTGGTCATCGGTC-3′	127 bp	Designed by WI state lab of hygiene for the purpose of wastewater surveillance
		NDM R	5′-CTGGCAGCACACTTCCTATCTCG-3′		
		NDM P	5′-HEX/AAGCGA+CT+GCCCCG+A+A+A+CCC/IABkFQ-3′		
Bacteroides human 16s rDNA *B. dorei*	qPCR	HF183F	5′-ATCATGAGTTCACATGTCCG-3′	86 bp	Designed to detect human Bacteroidales 16S rRNA gene in freshwater samples (67, 68)
		HF241R	5′-CGTTACCCCGCCTACTATCTAATG-3′		
		HF193p	5′-FAM/TCCGGTAGACGATGGGGATGCGTT/MGBNFQ-3′		
Clostridiales Group 2 assay Lachno3 16s rDNA V6 human	qPCR	Lachno3F	5′-CAACGCGAAGAACCTTACCAAA-3′	187 bp	Designed to detect Lachno3 marker in urban waters (69)
		Lachno3R	5′-CCCAGAGTGCCCACCTTAAAT-3′		
		Lachnop	5′-FAM/CTCTGACCGGTCTTTAATCGGA/MGBNFQ-3′		
*E. coli* β-glucuronidase	qPCR	uidA1663F	5′-GCGACCTCGCAAGGCATA-3′	127 bp	Designed to detect *E. coli* β-glucuronidase in water (70)
		uidA1790R	5′-GATTCATTGTTTGCCTCCCTGCTGCG-3′		
		uidA1729p	5′-VIC/TGCAGCAGAAAAGCCGCCGACTTCGG/MGBNFQ-3′		
*Klebsiella pneumoniae* hemolysin gene	qPCR	khe F	5′-CGATGCTACTTATCCCGACA-3′	78 bp	Designed for detection of the hemolysin gene of *K. pneumoniae* in fecal samples (71)
		khe R	5′-AGCCGGTTGAGACGTAAAC-3′		
		khe R	5′-HEX/CCGATTGAA/ZEN/AAACGCTCCGGGC/IABkFQ-3′		
*A. baumannii* biofilm-associated protein gene	qPCR	AB792 F	5′-CGCTGCAGCATCAAATCATG-3′	124 bp	Designed for detection of the biofilm-associated protein (bap) gene in whole blood samples (52)
		AB792 R	5′-AGGGTCAACCGAGAAAGTTACG-3′		
		AB792 P	5′-FAM/AGTACCTGC/ZEN/TGACACCACTCCACCA/3IABkFQ-3′		

^*a*^ All primers and probes in this table were purchased from IDT.

### Sample collection, filtration, and nucleic acid extraction

Wastewater influent samples were collected from two WWTPs that encompass the entirety of Milwaukee, WI (Table 2), as part of our ongoing Wisconsin SARS-CoV-2 wastewater surveillance program. This program has been part of the National Wastewater Surveillance System since August 2020 (72).

DNA gene target concentration and analysis for this paper includes a total of 122 untreated influent samples across the two WWTPs over the span of a year, September 2022 to August 2023, and an additional subset of 50 daily samples collected from JI and SS in July 2024. Flow-weighted composite samples were collected twice per week over a 24-h period from each WWTP, and aliquoted into 500 mL sterile Nalgene bottles by WWTP staff, delivered on ice, and held at 4°C for a maximum of 24 h prior to filtering 25 mL of raw wastewater, according to Feng et al. (73). HA filters were transferred to a 2 mL ZR BashingBead Lysis tube (Zymo Research, Catalog no. S6003-50) containing 500 μL MWA1 lysis buffer (MACHEREY-NAGEL) and held at −80°C for a minimum of 2 h prior to nucleic acid extraction. Extraction was performed using the NucleoMag DNA/RNA Water Kit (MACHEREY-NAGEL, Catalog no. 744220.1) with 400 μL of homogenized lysate on an automated instrument KingFisher Flex (Thermo Fisher Scientific). The 100 μL elution of each sample was held at −80°C until PCR-based or sequencing analysis. Extraction negative controls (no sample spike) were extracted using the same protocol. Detailed protocols on filtration and extraction are available on protocols.io (74).

### Sampling strategy

Specific subsets of the 122 samples, distributed evenly across SS and JI WWTPs (Fig. S7), were examined in this study to better understand the variability of ARG targets in the wastewater system and how they are impacted by various parameters in the sewer conveyance system. Samples included the following.

- A total of 50 daily samples over 25 days collected from 7 July 2024 to 31 July 2024, were analyzed to examine the precision of measurements and total variability of each ARG target and relationship to flow in each WWTP.
- A total of 72 samples collected were analyzed over the span of a year (7 September 2022–27 August 2023) to examine total variability and stability over time among changing conditions. Samples were collected twice per month, with a higher frequency of twice per week in the months with the coldest (April 2023) and warmest (August 2023) temperatures in the sewer system, to explore variability among different temperatures in the sewer system.
- A subset of 22 samples that were further analyzed for amplicon sequencing was performed on samples collected in the months of October, January, April (coldest), and August (warmest) (38) to assess variants detected by the assays and potential seasonal shifts.

### Droplet digital PCR

ddPCR was used to quantify six ARGs shown in Table 5. Sample DNA was diluted to levels within detection range, and ddPCR assays were performed according to the manufacturer’s instructions for a 22 μL reaction mixture using the ddPCR Supermix for Probes (Bio-Rad, Catalog no. 1863024) (Text S1). Reactions were amplified using the Mastercycler pro (Eppendorf), and read using the Bio-Rad QX200 Droplet Digital System as previously described (73).

Raw droplet amplification data were initially analyzed and extracted from the Bio-Rad QuantaSoft Analysis software and processed using R package twoddpcr (version 1.30.0) (75). All assays included a no-template control of nuclease-free water, and a positive control consisting of a ~350 bp gene fragment (Twist Biosciences) (Text S2). Positive control organisms are shown in Table S4. Inhibition was tested on all samples using bovine respiratory syncytial virus as described previously (73). None of the samples used in this study illustrated any signs of inhibition. Limits of blank, detection, and quantification are shown in Table S6 and described in Text S3.

The ddPCR variability was determined by calculating the percent difference on the same sample run on two separate ddPCR runs using the same conditions. A high abundance (*bla*_KPC_) and low abundance (*bla*_VIM_) ARG gene target were analyzed using JI and SS samples from July 2024. Percent average differences ranged from 17.7% to 23.8% for *bla*_VIM_ (*n* = 42) and *bla*_KPC_ (*n* = 46), respectively.

Sample variability was determined by calculating the percent difference on a filter created from two separate SS 24-h composite aliquots collected by the WWTP for a high abundance (*bla*_KPC_) and low abundance (*bla*_IMP_) target. Percent average variability ranged from 19.9% to 39.6% for *bla*_KPC_ (*n* = 16) and *bla*_IMP_ (*n* = 15), respectively.

### Quantitative PCR

Human markers Lachno3 and human *Bacteroides* HF183 were quantified using qPCR as previously described (39, 69). Additionally, markers for three host organisms *E. coli* (uidA), *Klebsiella pneumoniae* (KHE), and *Acinetobacter baumannii* (AB792) were analyzed via qPCR as indicated in Table 5. All reactions were performed according to the manufacturer’s instructions for a 25 μL reaction mixture using the 1× Taqman Gene Expression Master Mix (Applied Biosystems, Catalog no. 4369016), and amplifications were performed in a StepOnePlus Real Time PCR System (Applied Biosystems) as detailed in Text S4.

All samples were run in duplicate, and each assay included a no-template control of nuclease-free water and plasmid standard concentrations ranging from 1.5E+06 to 15 copies in 5 μL run in triplicate. The data were analyzed by the StepOnePlus software (version 2.3). Standard curve information is shown in Table S6. Limits of blank, detection, and quantification are described in Text S3.

Daily samples and an additional subset of 30 samples from the yearly subset confirmed that Lachno3 and HF183 were significantly correlated (Spearman rank correlation = 0.78 and 0.97, respectively), so this second human marker was not analyzed for the remainder of the data set.

### Amplicon sequencing

A subset of 22 samples was sequenced using forward and reverse primers of each target assay as described in Table 5 to confirm each respective assay performs specific amplification. PCRs were performed according to the manufacturer’s instructions for a 25 μL reaction using 1× HiFi HotStart Ready Mix (Roche Molecular Systems, Catalog no. 07958927001) as detailed in Text S5.

PCR products were purified, normalized using a Qubit 2 instrument (Thermo Fisher Scientific), size verified using a BioAnalyzer (Agilent Technologies), and pooled before dilution and loading onto the MiSeq DNA sequencer (Illumina). Raw reads were assessed for quality using FastQC (76), followed by adapter trimming in cutadapt (77), and read assembly with PEAR (78). ASVs were analyzed using the Dada2 pipeline in RStudio (79), then abundant ASVs were matched against NCBI’s nucleotide collection database (-db nr) using remote BLAST to determine associated taxa (80).

### 16S rRNA microbial community sequencing

All samples in the yearly subset, in addition to 18 samples upstream in the SS systems and 6 samples in the JI system, were analyzed for community analysis via 16S rRNA sequencing. PCR amplification of the V4V5 variable region of bacterial 16S rRNA gene was conducted using primers 518F and 926R (81). Samples were normalized using the Qubit 2 instrument (Thermo Fisher Scientific) and size verified using a BioAnalyzer (Agilent Technologies) before loading onto the MiSeq DNA sequencer (Illumina). A detailed protocol of 16S sequencing can be accessed on protocols.io (82). Sequences were trimmed, and low-quality reads were filtered out according to previously published methods (46).

### Community wastewater isolates

Wastewater from both JI and SS was diluted 1:2.5, and 20–30 μL was plated onto CHROMagar KPC (CHROMagar, Catalog no. KPRT2). Plates were incubated for 24 h, then a total of 50 colonies were selected based on presumed morphology (Table 6; Fig. S6). Colonies were boiled in 100 μL water, then 1:10 dilutions were run on all PCR assays for gene confirmation (Text S6). PCRs of the 16S gene (83) were sent to the University of Illinois Core Sequencing Facility to confirm bacterial species via Sanger sequencing (Text S7).

**TABLE 6:** The number of colonies selected for sequencing and PCR analysis, their morphology, and their expected bacterial type

Presumed bacteria type	Morphology	JI colonies	SS colonies
*E. coli*	Pink, purple	2	3
*Klebsiella*, *Enterobacter*, *Citrobacter*	Blue or purple/blue	9	12
*Acinetobacter*, *Pseudomonas*	Cream	8	10
Unknown	Yellow	3	3

### Statistical analysis

All data in this study were analyzed and reported as copies per liter of wastewater (cn/L) to account for dilutions, reaction volumes, and differing PCR methods. For analysis, samples that illustrated positive amplification below the LOQ were given a censored concentration of LOQ/2, and samples amplified below the LOD were given a censored concentration of LOD/2, taking into consideration the dilution of each reaction. A Shapiro-Wilk normality test determined that the use of Spearman’s rank correlation and non-parametric Wilcoxon test was the best method of analysis in this data set. Spearman’s rank correlation coefficients were used to measure the relationship between variables when exploring the correlations of different targets with one another. A Wilcoxon test was performed to determine whether flow or temperature significantly impacted ARG target concentration. Furthermore, all *P*-values were adjusted using the Benjamini-Hochberg procedure to control the false discovery rate (84).

All figures were created in R (version 4.4.1) or taken directly from Bio-Rad QuantaSoft software (version 1.7). Data were obtained directly from Bio-Rad QuantaSoft software, or the National Center for Biotechnology Information (NCBI) database, and were analyzed using R (version 4.4.1) (85).

## SUPPLEMENTAL MATERIAL

Supplemental material spectrum.01737-25-s0001.pdf Fig. S1 to S7; Tables S1 to S7; Texts S1 to S7; Data S1 and S2.

ASM does not own the copyrights to Supplemental Material that may be linked to, or accessed through, an article. The authors have granted ASM a non-exclusive, world-wide license to publish the Supplemental Material files. Please contact the corresponding author directly for reuse.
