# Supplementary material for: Environmental reservoirs account for high levels of carbapenem resistance genes in wastewater
Source: Microbiol Spectr. 2026 Jan 7;14(2):e01737-25. doi: 10.1128/spectrum.01737-25 (PMC12889080; doi:10.1128/spectrum.01737-25)
Supplement: Supplemental material — Fig. S1 to S7; Tables S1 to S7; Texts S1 to S7; Data S1 and S2. [file spectrum.01737-25-s0001.pdf]

Supplemental Information

Supplemental Figures

Figure S1. Amplitude shifts in ddPCR assays and associated variant base pair mismatches.
Figure S2. Distribution of BLAST hits for each ARG amplicon sequence type
Figure S3. Wilcoxon test of daily samples (July 2024) ARG concentration and daily average flow
Figure S4. Wilcoxon test of yearly samples (2022-2023) ARG concentration and daily average flow
Figure S5: Spearman's rank correlation matrix of all targets in each WWTP. All boxes not containing an "X" are significant ($p < 0.05$)
Figure S6: Morphology of colonies chosen for PCR and 16S sequencing analysis
Figure S7. Sampling strategy of wastewater samples for ddPCR analysis of five high priority carbapenem resistance genes and sequencing of the amplicon ARG products and the 16rRNA gene

Supplemental Tables

Table S1. NCBI blast results by percent frequency of the primers and probes matched to an organism for each assay listed in the Methods
Table S2. Enrichment of human and pipe-associated taxa in downstream WWTP samples compared with neighborhood samples determined by analysis of 16S rRNA microbial community data
Table S3. Spearman's correlation of 16S rRNA microbial community relative abundance data for select taxa to daily average temperature in SS and JI
Table S4. Detailed observations of bacterial isolates
Table S5. Clinical strains used for standards
Table S6. The standard curve information of all qPCR assays used in this study
Table S7. ddPCR assay limit of detection and limit of quantification values

Supplemental Text

Supplemental Text S1. Droplet Digital PCR (ddPCR)
Supplemental Text S2. Droplet Digital PCR (ddPCR) Standards, Validation, and quality controls
Supplemental Text S3. Limits of blank, detection, and quantification
Supplemental Text S4. Quantitative PCR (qPCR)
Supplemental Text S5. Amplicon Sequencing
Supplemental Text S6. Quick PCR of Community Wastewater Isolates
Supplemental Text S7. 16S Sequencing of Community Wastewater Isolates

Supplemental Data Sets

Supplemental Data Set S1. Master data containing all samples analyzed in this paper
Supplemental Data Set S2. Gene fragment sequences used for ddPCR standards

Supplemental Figures

A.

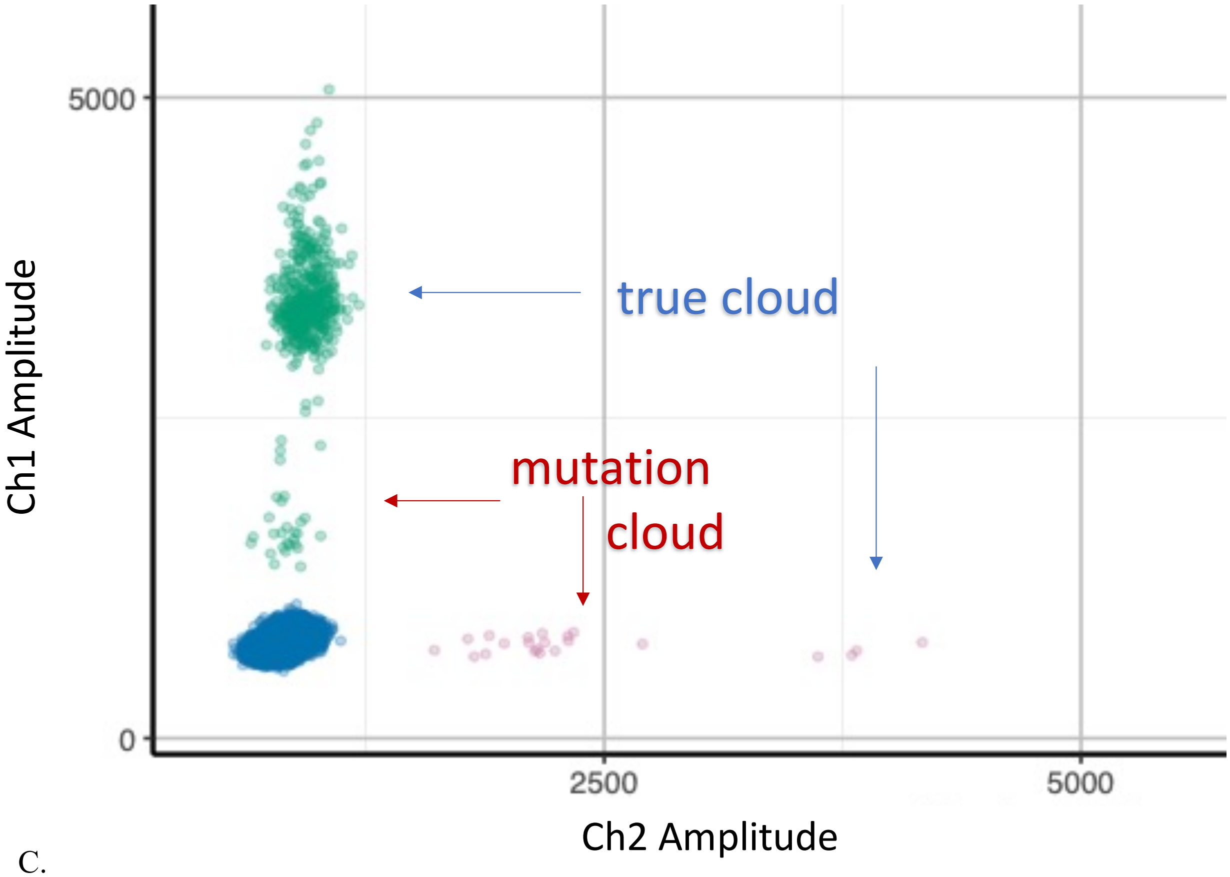

C.

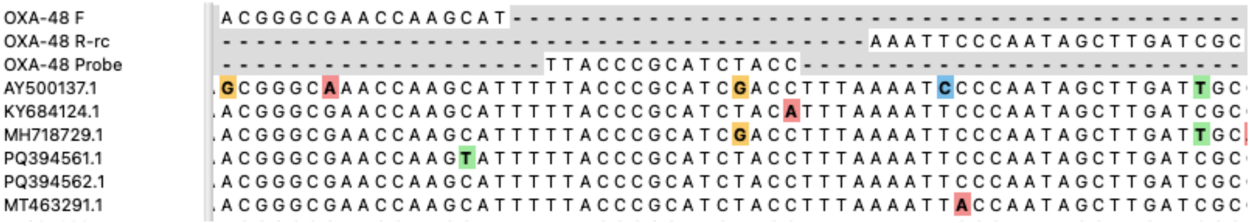

B.

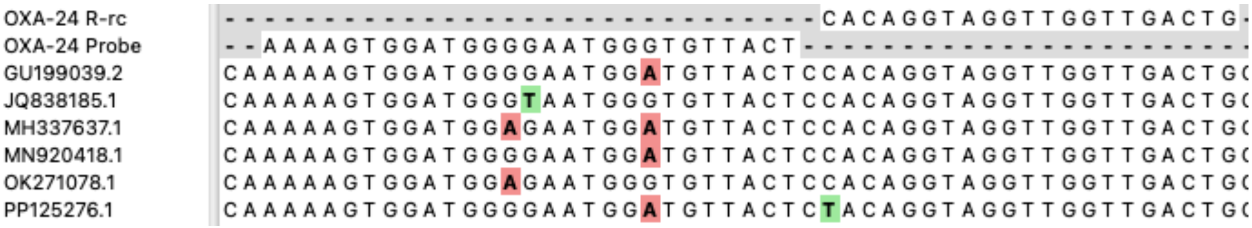

Figure S11. Amplitude shifts in ddPCR assays and associated variant base pair mismatches. A. Double cloud formation of both $\textit{bla}_{\text{OXA-48}}$ (Ch. 1 amplitude) and $\textit{bla}_{\text{OXA-24/40}}$ (Ch. 2 amplitude) assays. B. $\textit{bla}_{\text{OXA-48}}$ group aligned sequences with probe mismatches: AY500137.1 ($\textit{bla}_{\text{OXA-54}}$), KM589641.1 ($\textit{bla}_{\text{OXA-405}}$), KY684124.1 ($\textit{bla}_{\text{OXA-547}}$). C. $\textit{bla}_{\text{OXA-24/40}}$ group aligned sequences with probe mismatches: OK271078.1 (*bla*OXA-1040), PP125276.1 (blaOXA1225), JQ838185.1 ($\textit{bla}_{\text{OXA-207}}$), MH337637.1 ($\textit{bla}_{\text{OXA-653}}$), GU199039.2 ($\textit{bla}_{\text{OXA-72}}$), MN920418.1 ($\textit{bla}_{\text{OXA-897}}$).

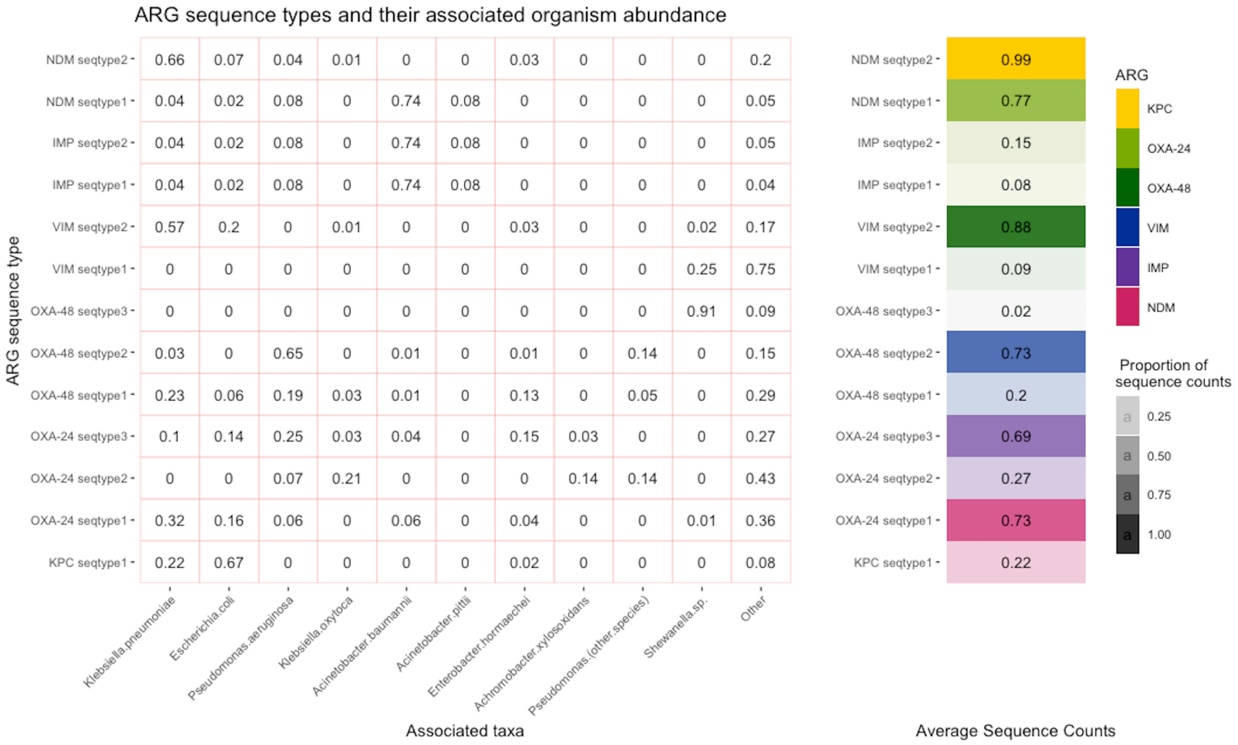

Figure S2. Distribution of BLAST hits for each ARG amplicon sequence type. The relative abundance for each sequence type within a targeted ARG is shown in the legend and corresponds to averages across samples from Figure 2. Numbers in the table correspond to the proportion of BLAST hits for each organism for a particular sequence type (shown as rows).

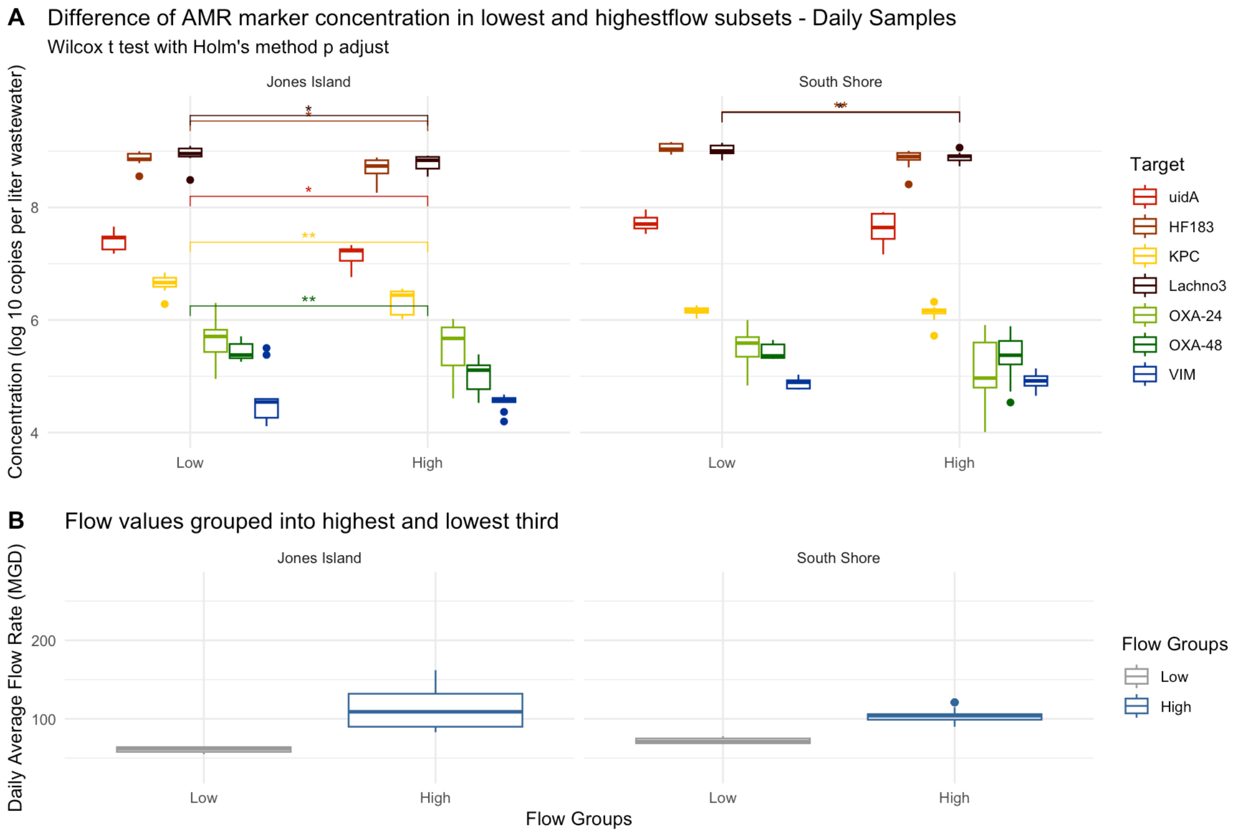

Figure S3. Box plot of concentrations under the highest (upper 1/3 of measurements) and lowest (lower 1/3) flow conditions (n = 9 per group) for the daily sample subset in JI and SS WWTP. A "*" above a bracket indicates significance ($p < 0.05$) to concentration of each target under high or low flow conditions, with each additional "*" representing an additional order of magnitude. Box plots containing no brackets are not significant. B. Box plot depicting the range in daily average flow values (MGD) in the high and low subsets (n = 9) in JI and SS WWTP. High flow and low flow groups used in this analysis were significantly different (Wilcoxon test, $p < 0.0001$). The targets bla_{IMP} and bla_{NDM} were not analyzed due to several sample concentrations being below the limits of quantification for these targets.

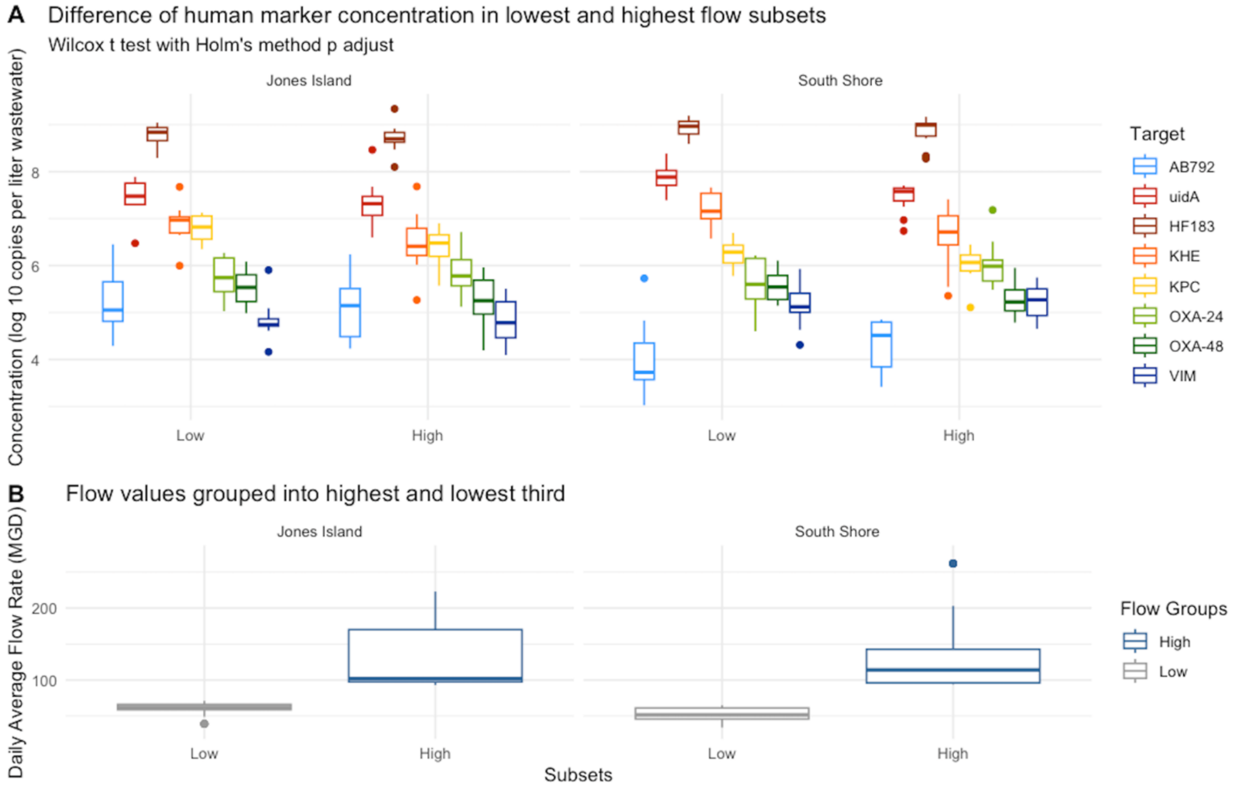

Figure S4. Box plot of concentrations under the highest and lowest flow conditions (n =12 per group) for all samples across the 2022-2023 yearly samples in JI and SS WWTP. A "*" above a bracket indicates significance ($p < 0.05$) to concentration of each target under high or low flow conditions. Box plots containing no brackets are not significant. B. Boxplot depicting the range in daily average flow values (MGD) in the high and low subsets (n = 12) in JI and SS WWTP. High flow and low flow groups used in this analysis were significantly different (Wilcoxon test, $p < 0.0001$).

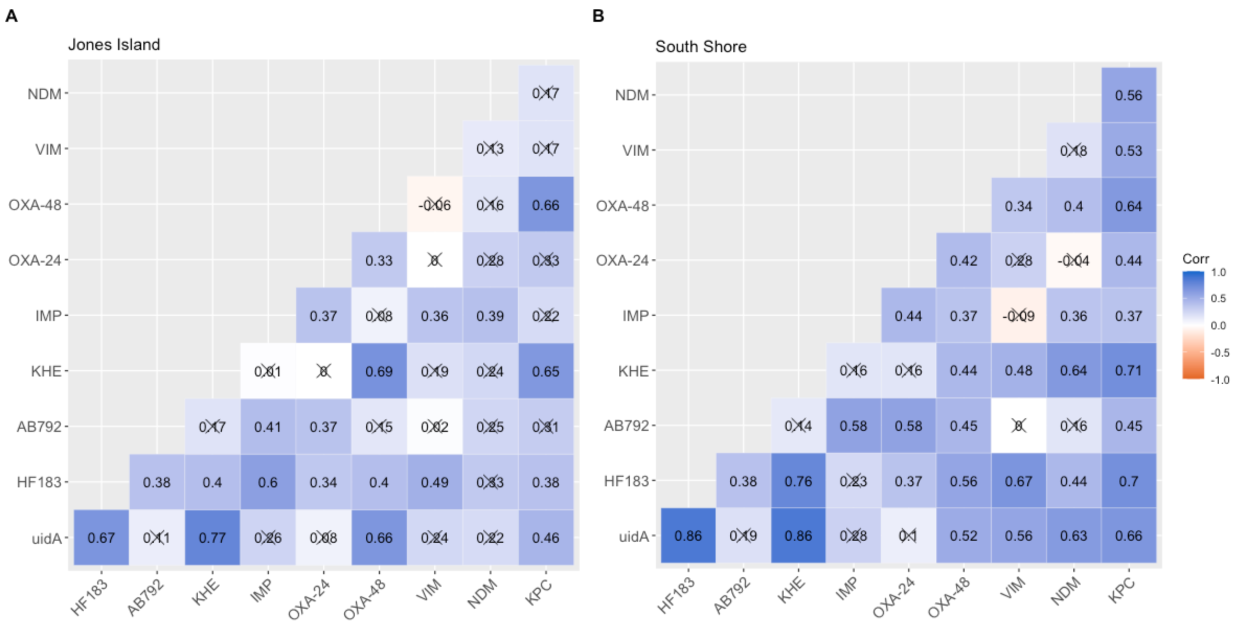

Figure S5. Spearman's rank correlation matrix of all gene targets in Jones Island (A) and South Shore (B). All boxes not containing an "X" are significant ($p < 0.05$).

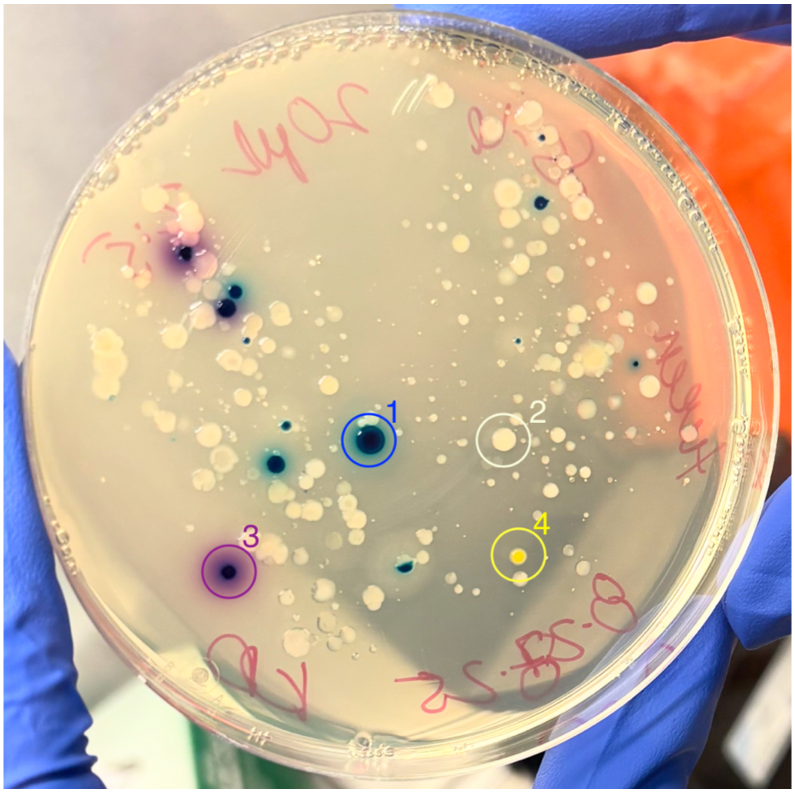

Figure S6: Morphology of colonies chosen for PCR and 16S sequencing analysis. 1. Presumed *Klebsiella, Enterobacter*, or *Citrobacter* (KEC) represented by a metallic blue color. 2. Presumed *Acinetobacter*, represented by an opaque cream color. 3. Presumed *E. coli* represented by a dark pink/ purple color. 4. Unknown bacterial type represented by bright yellow color.

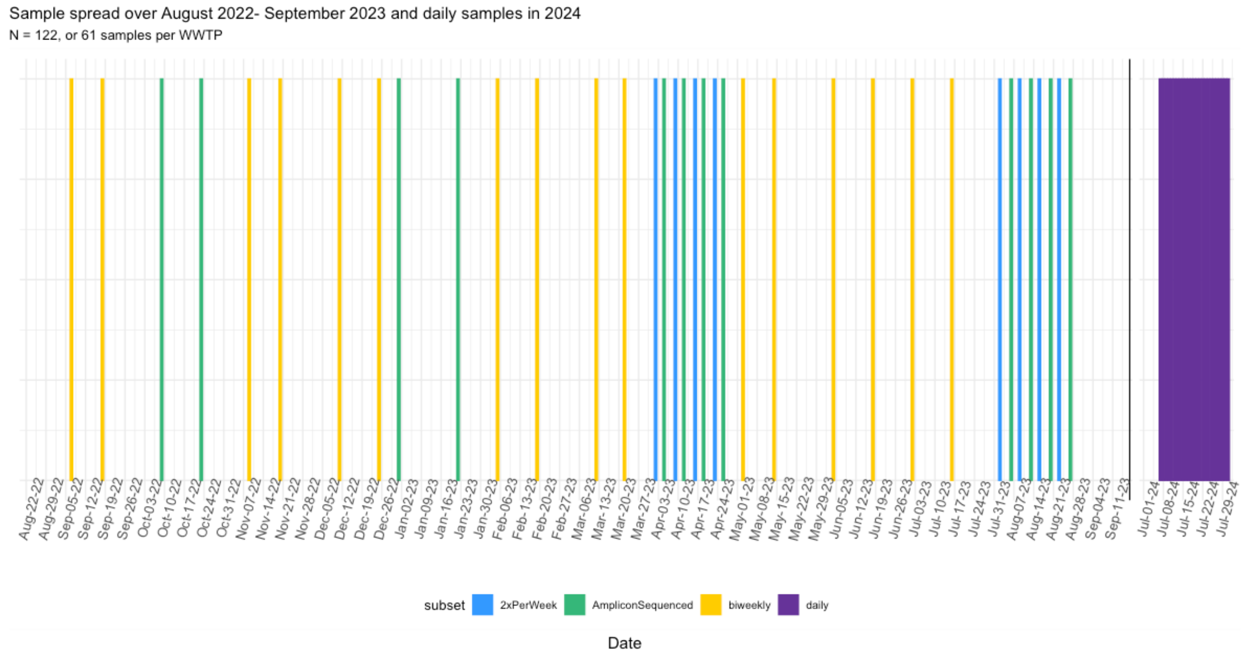

Figure S7. Sampling strategy of wastewater samples for ddPCR analysis of five high priority carbapenem resistance genes and sequencing of the amplicon ARG products and the 16rRNA gene. The yellow bands represent samples that were collected twice per month from September 2022 to August 2023. The blue bands represent samples collected twice per week in April and August to account for seasonal shifts. Samples indicated by green bands were further analyzed for amplicon sequencing. The purple bands represent samples collected daily in July 2024.

Supplemental Tables

Table S1. NCBI BLAST taxa results by percent frequency for the primers and probes used in each assay listed in the methods (Table 5).

BLAST of Primers & Probe

***bla*$_{KPC}$ Taxa**	**% Frequency**
Klebsiella pneumoniae	65
Escherichia coli	7
Pseudomonas aeruginosa	4
***bla*$_{OXA-24/40}$ Taxa**	**% Frequency**
Acinetobacter baumannii	93
Acinetobacter nosocomialis	4
Acinetobacter haemolyticus	2
***bla*$_{OXA-48}$ Taxa**	**% Frequency**
Klebsiella pneumoniae	55
Escherichia coli	19
uncultured bacterium	4
***bla*$_{VIM}$ Taxa**	**% Frequency**
Pseudomonas aeruginosa	43
Klebsiella pneumoniae	11
Enterobacter hormaechei	4
***bla*$_{IMP}$ Taxa**	**% Frequency**
Pseudomonas aeruginosa	24
Escherichia coli	15
Enterobacter hormaechei	14
***bla*$_{NDM}$ Taxa**	**% Frequency**
Escherichia coli	32
Klebsiella pneumoniae	28
Pseudomonas aeruginosa	4

Table S2. Enrichment of human and pipe-associated taxa at the WWTP compared with upstream conveyance system sites determined by analysis of 16S rRNA microbial community data (n = 72).

Taxa[1]	No. of ASVs[2]	South Shore		Jones Island	
		No. reads	fold enrichment[3]	No. reads	fold enrichment[3]
Acinetobacter	23	8595	1.8x	13952	2.8x
A. baumannii	1	6	-1.3x	19	2.0x
Aeromonas	3	3701	4.0x	3257	2.6x
Arcobacter	17	4852	5.6x	3302	1.7x
Bacteroides (V4V5-1)	1	311	2.0x	289	2.0x
Enterobacter	1	330	3.4x	319	3.0x
Escherichia	1	100	-1.7x	51	-3.2x
Klebsiella	2	431	4.1x	389	6.4x
*Klebsiella*_A	1	123	5.1x	126	5.2x
*Pseudomonas*_E	27	1070	-1.3x	1543	1.9x
Shewanella	1	29	2.1x	9	1.2x
Enterobacteriaceae NA	5	160	2.2x	408	4.0x
*Bacteroides*_B (HF183)	1	280	-5.5x	231	1.0x
*Blautia*_A	10	1382	-3.2x	1550	-2.8x

[1] Genera are grouped into three subsets: sewer residents, fecally shed bacteria known to persist within the environment, and human fecal bacteria.
[2] Average across all samples for ASVs within each taxa with samples normalized to 100,000 sequence reads per sample
[3] Fold enrichment indicates the fold increase or decrease (represented by negative values) in the relative abundance of taxa in the WWTP compared with the upstream neighborhood.

Table S3. Spearman's correlation of 16S rRNA microbial community relative abundance data for select taxa to daily average temperature in SS and JI WWTP. Genera are grouped into three subsets: sewer residents, fecally shed bacteria known to persist within the environment, and human fecal bacteria.

	Jones Island		South Shore	
**Genus**	**R value**	**p-value**	**R value**	**p-value**
**Acinetobacter**	**-0.132**	**0.0001**	**-0.113**	**0.0004**
Aeromonas	0.074	0.4610	-0.027	0.7810

Arcobacter	0.045	0.2630	-0.023	0.5520
**Bacteroides ASV[1]**	**0.747**	**<0.0001**	**0.830**	**<0.0001**
**Enterobacter**	**0.469**	**0.0051**	**-0.451**	**0.0057**
**Escherichia**	**0.644**	**<0.0001**	**0.733**	**<0.0001**
**Klebsiella**	**0.417**	**0.0004**	**0.412**	**0.0003**
**Klebsiella_A**	**0.668**	**<0.0001**	**0.601**	**0.0001**
**Pseudomonas_E**	**-0.109**	**0.0015**	**-0.111**	**0.0008**
Shewanella	-0.063	0.7217	-0.171	0.3185
**Enterobacteriaceae**	-0.102	0.2353	**-0.242**	**0.0035**
Bacteroides_B[2]	0.268	0.1250	0.329	0.0504
**Blautia_A**	0.018	0.7300	**0.125**	**0.0130**

[1] A single ASV representing an environmentally occurring Bacteroides was used in this analysis

[2] A single ASV representing the HF183 markers was used in this analysis

Table S4. Detailed observations of bacterial isolates

**Target**	**WWTP**	**Presumptive ID[1]**	**Morphology**	**Detected PCR gene**	**identity**	**16S Bacteria**
EC10	JI	E. coli	dark and shiny	***bla***$_{KPC}$	0.990	Enterobacteriaceae
EC20	SS	E. coli	shiny, small, flat, mucoid	***bla***$_{KPC}$	0.978	Citrobacter
EC3	JI	E. coli	purple with dark blue center	***bla***$_{KPC}$	0.998	Gammaproteobacteria
KPC1	JI	KEC	round & shiny, no mucoid	***bla***$_{KPC}$	0.978	Aeromonas
KPC10	JI	KEC	round & shiny, no mucoid	***bla***$_{KPC}$	0.995	Aeromonas
KPC13	JI	KEC	round and shiny	***bla***$_{KPC}$	0.989	Aeromonas
KPC17	JI	KEC	round and shiny	***bla***$_{KPC}$	0.983	Aeromonas
KPC20	JI	KEC	flat and shiny	***bla***$_{KPC}$	0.997	Aeromonas
KPC28	SS	KEC	big, shiny, raised	***bla***$_{KPC}$	0.970	Aeromonas
KPC3	JI	KEC	round & shiny, no mucoid	***bla***$_{KPC}$	0.986	Aeromonas
KPC31	SS	KEC	flat and shiny	***bla***$_{KPC}$	0.984	Aeromonas
KPC32	SS	KEC	flat and shiny	***bla***$_{KPC}$	0.989	Aeromonas
KPC33	SS	KEC	flat and shiny	***bla***$_{KPC}$	0.993	Aeromonas
KPC34	SS	KEC	flat and shiny	***bla***$_{KPC}$	0.999	Aeromonas
KPC36	SS	KEC	flat and shiny	***bla***$_{KPC}$	0.986	Aeromonas

KPC40	SS	KEC	flat and shiny	***bla*_KPC_**	0.993	Aeromonas
KPC6	JI	KEC	round & shiny, no mucoid	***bla*_KPC_**	0.996	Aeromonas
KPC8	JI	KEC	round & shiny, no mucoid	***bla*_KPC_**	0.993	Aeromonas
A10	JI	Acinetobacter	uneven edges, center & ring, larger	NA	0.994	Pseudomonas
A11	SS	Acinetobacter	small, round, flat	NA	0.998	Pseudomonas
A12	SS	Acinetobacter	large, raised, shiny, mucoid	NA	0.997	Pseudomonas
A13	SS	Acinetobacter	shiny, round, raised	NA	0.995	Pseudomonas
A14	SS	Acinetobacter	like A2 but larger and with smoother edges	NA	0.948	Pseudomonas
A15	SS	Acinetobacter	small, round, flat	NA	0.997	Comamonas
A16	SS	Acinetobacter	shiny, flat, circular	NA	0.997	Pseudomonas
A17	SS	Acinetobacter	shiny, flat, circular	NA	0.996	Pseudomonas
A18	SS	Acinetobacter	shiny, flat, circular, mucoid	NA	0.999	Pseudomonas
A19	SS	Acinetobacter	shiny, flat, circular	NA	0.996	Pseudomonas
A2	JI	Acinetobacter	uneven edges, center & ring	NA	0.997	Pseudomonas
A20	SS	Acinetobacter	shiny, flat, circular, large	NA	0.996	Pseudomonas
A3	JI	Acinetobacter	circular, smooth edges	NA	0.994	Pseudomonas
A4 - 8F	JI	Acinetobacter	circular, smooth edges	NA	1.000	Pseudomonas
A5	JI	Acinetobacter	circular, smooth edges	NA	0.999	Pseudomonas
A6	JI	Acinetobacter	circular, smooth edges	***bla*_OXA-24/40_**	0.976	Acinetobacter
A7	JI	Acinetobacter	uneven edges, center & ring	NA	0.999	Pseudomonas
A9	JI	Acinetobacter	mix of smooth and uneven edges	NA	0.999	Pseudomonas
EC14	SS	E. coli	dark, shiny, and raised	NA	0.999	Enterobacteriaceae
EC16	SS	E. coli	dark, shiny, flat, circular	NA	1.000	Enterobacteriaceae
KPC18	JI	KEC	flat and shiny	NA	0.994	Aeromonas
KPC25	SS	KEC	shiny and flat	NA	0.996	Aeromonas
KPC30	SS	KEC	shiny and flat	NA	0.999	Aeromonas
KPC35	SS	KEC	flat and shiny	NA	0.997	Aeromonas
KPC37	SS	KEC	small, shiny, raised	NA	0.994	Aeromonas
KPC38	SS	KEC	big, flat, shiny	NA	0.999	Aeromonas
Y1	SS	Pseudomonas	small, round, shiny, flat	***bla*_OXA-24/40_**	0.999	Pseudomonas
Y2	SS	Pseudomonas	small, round, shiny, flat	NA	0.997	Pseudomonas
Y3	SS	Pseudomonas	small, round, shiny, flat	NA	0.998	Pseudomonas
Y4	JI	Pseudomonas	small, round, shiny, flat	NA	0.998	Pseudomonas
Y5	JI	Pseudomonas	small, round, shiny, flat	NA	0.999	Pseudomonas
Y6	JI	Pseudomonas	small, round, shiny, flat	NA	0.999	Pseudomonas

[1]KEC *Klebsiella*, *Enterobacter*, or *Citrobacter*

Table S5. Clinical strains used for standards on ddPCR.

CDC AR Bank #	Bacteria	Resistance Gene Variant
AR 116 *	Citrobacterfreundii	KPC-2
AR 101 (nt)	Acinetobacter baumannii	OXA-24/40
AR 160 *	Klebsiella pneumoniae	OXA-48
AR 154 *	Enterobacter cloacae	VIM-1
AR 161 *	Klebsiella aerogenes	IMP-4
AR 033	Acinetobacter baumannii	NDM-1

Table S6. The standard curve information of all qPCR assays used in this study

	HF183	uidA	Lachno3	KHE	AB792
**Slope**	-3.41	-3.37	-3.42	-3.274	-3.174
**Y-intercept**	38.72	37.14	38.13	36.096	35.713
**R2**	1.00	1.00	1.00	0.999	0.998
**Eff%**	96.72	98.18	95.92	102.061	106.581

Table S7. LOB, LOD, and LOQ values with each associated ARG target detected on ddPCR. Values were calculated as droplets number. Corresponding copy number was calculated according to the average concentration of 1 droplet per reaction (0.077cp/μL).

Target	LOB	LOD	LOD cp/μL	LOQ	LOQ cp/μL
bla_{KPC}	1	2	0.154	5	0.386
$bla_{OXA\text{-}24/40}$	0	1	0.077	6	0.463
$bla_{OXA\text{-}48}$	2	2	0.154	11	0.848
bla_{VIM}	0	1	0.077	10	0.771
bla_{IMP}	0	1	0.077	6	0.463
bla_{NDM}	1	1	0.077	11	0.848

Supplemental Text

Supplemental Text S1. Droplet Digital PCR (ddPCR)

bla$_{OXA-24/40}$ and *bla*$_{OXA-48}$ were the only ARG targets run as a duplex, we obtained similar results when running *bla*$_{OXA-24/40}$ and *bla*$_{OXA-48}$ separately. This assay was run starting at 1:100 due to the multiple amplification clouds (Figure S1), as positive droplets were easier to distinguish when strongly diluted. *Bla*$_{KPC}$, *bla*$_{VIM}$ were run starting at a dilution of 1:50, *bla*$_{IMP}$ and *bla*$_{NDM}$ were run at a dilution starting at 1:10. If any samples were below LOQ, dilutions were lowered as necessary and available.

All reactions were made up using a mixture consisting of 1X ddPCR Supermix for Probes (No dUTP) (Bio-Rad, Hercules, CA, USA), 900 nM forward and reverse primers, 250 nM Taqman probe, 5.5 μL of the diluted DNA extract, and DNase-free water to get the total reaction volume of 22 μL.

The thermal cycling conditions were 10 min at 95°C followed by 40 cycles of 94°C for 30 sec, optimal annealing temp for 1 min: 53°C for *bla*$_{IMP}$, 54°C for the *bla*$_{OXA}$ duplex, 58°C for *bla*$_{KPC}$, 60°C for *bla*$_{NDM}$ and *bla*$_{VIM}$. Annealing was followed by a single deactivation step of 10 min at 98°C, and droplet stabilization for a minimum of 30 min at 4°C. Temperature gradients were performed to confirm the optimal annealing temperature by observing the clearest and largest separation between the positive and negative droplet amplitudes (1).

Supplemental Text S2. Droplet Digital PCR (ddPCR) Standards, Validation, and quality controls

Alignments of primers and probes were performed using the MegAlign Pro program in DNAStar software (Version 17.3.0.57) with corresponding host organisms of each ARG. Linear synthesized DNA fragments (Twist Biosciences) were made using a sequence of approximately 350 bp surrounding the alignment for PCR standard controls. All gene fragments were diluted 1:10000000 (1 ng/μL) prior to use as a standard on ddPCR.

Bacterial positive controls were obtained from the CDC & FDA Antibiotic Resistance Isolate Bank (https://wwwn.cdc.gov/ARIsolateBank/Search) (Table S5) to confirm that gene fragments and assays operate as expected. Gene fragments and CDC bacteria isolates were run on gels to confirm our assays are picking up the correct target using 1X PCR Master Mix (Promega, Catalog no. M7502, Madison, WI, USA), on 2.5% agarose gel, and on ddPCR to confirm we are examining the correct amplitude.

Positive controls (standards) and no template control (NTC) reactions were used to guide the threshold position(s) following the guidelines published in (2). Reactions were considered to have passed quality control if the droplet count was greater than 10,000, the NTC on each reaction was below the LOD (Table S7), and the standards on each reaction illustrated a clear band at the desired amplitude. No samples in this analysis failed these quality control measures. Additionally, all samples passed inhibition control consisting of spiking a known amount of

Bovine Respiratory Syncytial Virus (approximately 4,000 copies) into each sample, and then quantifying by ddPCR according to Feng et al (3).

Supplemental Text S3. Limits of blank, detection, and quantification

The limit of blank (LOB) is defined as the maximum concentration expected in a blank sample. For all ddPCR assays, the LOB was calculated according to the NCCLS guideline of EP17-A (4), obtaining the 95% confidence interval across 30 replicate DNAase-free water samples.
We defined the limit of detection (LOD) as the lowest analyte concentration likely to be reliably distinguished from the LOB. To determine this value, we took the 95th percentile for the standard deviation of all low-level samples (detected below 1 cp/µL) and added that to the LOB. This value was rounded up to the nearest higher integer to preserve a conservative value.
We define the LOQ as the lowest concentration of the analyte for which the method provides results with an acceptable uncertainty (5). To determine this, serial 1:2 dilutions of clinical isolates pertaining to the target gene of each assay were run in duplicate, the LOQ was set to the concentration at which the relative standard deviation was less than or equal to 30% between duplicate measurements, and the difference between the calculated and expected concentrations was less than 30%. All LOB, LOD, and LOQ values are described in Table S7.

For all qPCR assays, the LOD is defined as the last linear point on the standard curve (Ct = 35), meaning any samples amplified at a cycle number above 35 were considered below LOD. Samples that amplify with a Ct lower than 35, but with a quantity mean of less than 15 copies per reaction, were considered below LOQ.

Supplemental Text S4. Quantitative PCR (qPCR)

Samples were diluted 1:10 for AB792, 1:50 for KHE, and 1:100 for HF183, Lachno3, and uidA prior to spiking into reaction mixture consisting of 1X Taqman Gene Expression Master Mix (Applied Biosystems, Foster City, CA, USA), 1 µM forward and reverse primers (Integrated DNA Technologies), 80 nM Taqman probe (Applied Biosystems), 5 µL of the diluted DNA extract, and dnase-free water to get the total reaction volume of 25 µL.

The thermal cycling conditions were 2 min at 50°C, 10 min at 95°C followed by 40 cycles of 95°C for 15 sec and 1 min at an optimal annealing temperature: 60°C for HF183, uidA, and KHE, 63°C for AB792, and 64°C for Lachno3. Temperature gradients were performed to confirm the optimal annealing temperature by observing at which temperature produced the strongest DNA concentration product on a gel.

Standard curve and efficiency information from each assay are indicated in Table S2. Quantitative genomic DNA Acinetobacter baumannii (ATCC, Catalog no. 17978DQ), and Klebsiella pneumoniae subsp. pneumoniae (ATCC, Catalog no. 700721DQ) were used to make AB792 and KHE standards on RT-qPCR.

Each group of samples is run with a full standard curve in triplicate (1.5E6 to 1.5E1) for the first full plate. Each successive plate is run with 3 standards (1.5E3 to 1.5E5), and the quantity is

calculated for all plates using a yearly average slope and Y intercept. No template controls (NTC) are used to check for contamination. Reactions were only accepted if the NTC resulted in a Ct of 0.

Supplemental Text S5. Amplicon Sequencing

Amplification was performed using a 25 µL reaction using 1X HiFi HotStart Ready Mix (Roche, Catalog no. 7958935001), 300 nM of each primer, and 5 µL diluted DNA. For sequencing, assays were diluted at various amounts depending on the strength of DNA concentration detected in each reaction using a Qubit 2.0 Fluorometer (Thermo Fisher Scientific). Samples were diluted according to use for which assay: bla_{KPC} was diluted 1:5, $bla_{OXA\text{-}24/40}$ was diluted 1:100, $bla_{OXA\text{-}48}$ was diluted 1:5, bla_{VIM} was diluted 1:50, bla_{IMP} was diluted 1:10, and bla_{NDM} was diluted 1:6. The thermal cycling conditions were 5 min at 95°C followed by 35 cycles of 98°C for 8 seconds, optimal annealing temp for 1 minute (bla_{KPC}, 58°C for $bla_{OXA\text{-}24/40}$, $bla_{OXA\text{-}48}$, and bla_{VIM}, 53°C for bla_{IMP}, and 65°C for bla_{NDM}), and 20 seconds at 72°C. A final deactivation step of 1 min at 72°C before cooling the reaction to and holding at 4°C. All annealing temperatures were determined by performing a temperature gradient, and selecting the temperature that produced the highest yield of DNA while producing a band at the appropriate size on agarose gel.

PCR products were purified using a double-sided Agencourt Ampure XP (Beckman Coulter, Catalog no. A63882) SPRI bead purification for products 97 base pairs and larger or a right-side Agencourt Ampure XP purification combined with Small Fragment Extraction kit (IBI Scientific, Catalog no. IB47061) to retain products less than 100 base pairs. xGen DNA Library Prep Kit MC UNI (Integrated DNA Technologies, Catalog no. 10009820) was used to prepare all PCR products for next generation sequencing. PCR products underwent end-repair and dA-tailing in a single end prep reaction, followed by ligation of full-length xGen UDI-UMI adapters. Ligation reactions were purified using Agencourt Ampure XP SPRI beads, and the fully indexed libraries were amplified with 3 PCR cycles.
Resulting libraries were quantified using a Qubit 2 Fluorometer (Thermal Fisher Scientific), size verified using a BioAnalzyer (Agilent), normalized and pooled before dilution and denaturation. The denatured library was diluted to 8 pM, combined with 8 pM PhiX, and loaded onto an Illumina MiSeq DNA sequencer using the 500-cycle kit (Illumina, catalog no. MS-102-2003).

Supplemental Text S6. Quick PCR of Community Wastewater Isolates

Confirmation of genes was performed by using a reaction mixture consisting of 1X Taqman Gene Expression Master Mix (Applied Biosystems, Foster City, CA, USA), 1 µM forward and reverse primers (Integrated DNA Technologies), 80 nM Taqman probe (Applied Biosystems), 2 µL of the diluted boiled colonies, and dnase-free water to get the total reaction volume of 10 µL. The thermal cycling conditions were as previously described in Supplemental Text S1- S5. Each plate contained one positive standard and one NTC to rule out a failed amplification or contamination. All samples with a Ct above 15, but below 35, were considered positive for harboring the retrospective gene.

Supplemental Text S7. 16S Sequencing of Community Wastewater Isolates

PCR was performed using a reaction mixture consisting of 12.5 µL PCR Master Mix (Promega, catalog no. M7502), 1 µM 8F and 1492R primers (Integrated DNA Technologies) (6), 3 µL of undiluted boiled colonies, and DNase-free water to get the total reaction volume of 25 µL. The thermal cycling conditions were 3 min at 95°C followed by 34 cycles of 95°C for 30 seconds, 55°C for 30 seconds, and 60 seconds at 72°C. A final deactivation step of 5 min at 72°C before cooling the reaction to and holding at 10°C, until samples were ready for PCR clean-up.

All reactions were analyzed on a 1% agarose gel with Low DNA Mass Ladder (Thermo Fisher Scientific, catalog no. 10068013), and primers were removed using a standard 1X SPRI bead clean up protocol (Omega Bio-Tek, catalog no. M1378-01). Cleaned-up samples were shipped on ice to the University of Illinois at Urbana-Champaign Roy J. Carver Biotechnology Center Core Sequencing Facility (https://corelims.biotech.illinois.edu), along with 10 pmol 8F and 1492R primers for Sanger sequencing. Returned trace files were checked for quality by examining the chromatograms, and were transferred to FASTQ files using abi2fastq (7). The FASTQ files of the same sample were then merged using PEAR (8) to assembled full length 16S sequences. The sequences were then classified for taxonomy using MAPSeq (9) with reference to Silva database.

References:

1. Maar D, Prantner A. 2020. Transitioning Your Assay from Quantitative PCR to Droplet Digital PCR. Bio-Rad Bull 7320.

2. Decaro N, Elia G, Campolo M, Desario C, Mari V, Radogna A, Colaianni ML, Cirone F, Tempesta M, Buonavoglia C. 2008. Detection of bovine coronavirus using a TaqMan-based real-time RT-PCR assay. J Virol Methods 151:167–171.

3. Feng S, Roguet A, McClary-Gutierrez JS, Newton RJ, Kloczko N, Meiman JG, McLellan SL. 2021. Evaluation of Sampling, Analysis, and Normalization Methods for SARS-CoV-2 Concentrations in Wastewater to Assess COVID-19 Burdens in Wisconsin Communities. ACS EST Water 1:1955–1965.

4. Moretti M, Sisti D, Rocchi MB, Delprete E. 2011. CLSI EP17-A protocol: A useful tool for better understanding the low end performance of total prostate-specific antigen assays. Clin Chim Acta 412:1143–1145.

5. Deprez L, Corbisier P, Kortekaas A-M, Mazoua S, Beaz Hidalgo R, Trapmann S, Emons H. 2016. Validation of a digital PCR method for quantification of DNA copy number concentrations by using a certified reference material. Biomol Detect Quantif 9:29–39.

6. Turner S, Pryer KM, Miao VPW, Palmer JD. 1999. Investigating Deep Phylogenetic Relationships among Cyanobacteria and Plastids by Small Subunit rRNA Sequence Analysis1. J Eukaryot Microbiol 46:327–338.

7. Botvinnik O. 2017. abi2fastq (1.0.4).

8. Zhang J, Kobert K, Flouri T, Stamatakis A. 2014. PEAR: a fast and accurate Illumina Paired-End reAd mergeR. Bioinformatics 30:614–620.

9. Matias Rodrigues JF, Schmidt TSB, Tackmann J, Von Mering C. 2017. MAPseq: highly efficient k-mer search with confidence estimates, for rRNA sequence analysis. Bioinformatics 33:3808–3810.
